# CDK4/6 inhibitors and the pRB-E2F1 axis suppress PVR and PD-L1 expression in triple-negative breast cancer

**DOI:** 10.1038/s41389-023-00475-1

**Published:** 2023-05-26

**Authors:** Mariusz Shrestha, Dong-Yu Wang, Yaacov Ben-David, Eldad Zacksenhaus

**Affiliations:** 1 Department of Laboratory Medicine & Pathobiology, University of Toronto, Toronto, Ontario Canada; 2 Toronto General Research Institute - University Health Network, 101 College Street, Max Bell Research Centre, Rm. 5R406, Toronto, Ontario M5G 1L7 Canada; 3 The Key Laboratory of Chemistry for Natural Products of Guizhou Province and Chinese Academic of Sciences, 550014 Guiyang, Guizhou China; 4 State Key Laboratory for Functions and Applications of Medicinal Plants, Guizhou Medical University, 550025 Guiyang, China

**Keywords:** Breast cancer, Tumour immunology

## Abstract

Immune-checkpoint (IC) modulators like the poliovirus receptor (PVR) and programmed death ligand 1 (PD-L1) attenuate innate and adaptive immune responses and are potential therapeutic targets for diverse malignancies, including triple-negative breast cancer (TNBC). The retinoblastoma tumor suppressor, pRB, controls cell growth through E2F1-3 transcription factors, and its inactivation drives metastatic cancer, yet its effect on IC modulators is contentious. Here, we show that RB-loss and high E2F1/E2F2 signatures correlate with expression of *PVR*, *CD274* (PD-L1 gene) and other IC modulators and that pRB represses whereas RB depletion and E2F1 induce *PVR* and *CD274* in TNBC cells. Accordingly, the CDK4/6 inhibitor, palbociclib, suppresses both PVR and PD-L1 expression. Palbociclib also counteracts the effect of CDK4 on SPOP, leading to its depletion, but the overall effect of palbociclib is a net reduction in PD-L1 level. Hydrochloric acid, commonly used to solubilize palbociclib, counteracts its effect and induces PD-L1 expression. Remarkably, lactic acid, a by-product of glycolysis, also induces PD-L1 as well as PVR. Our results suggest a model in which CDK4/6 regulates PD-L1 turnover by promoting its transcription via pRB-E2F1 and degradation via SPOP and that the CDK4/6-pRB-E2F pathway couples cell proliferation with the induction of multiple innate and adaptive immunomodulators, with direct implications for cancer progression, anti-CDK4/6- and IC-therapies.

## Introduction

During cancer progression, tumor cells employ diverse strategies to escape immune surveillance by, for example, overexpressing programmed death ligand 1 (PD-L1; cluster of differentiation 274, *CD274*), which binds programmed cell death protein 1 (PD-1, *PDCD1*) on the surface of cytotoxic CD8+ T cells and attenuates their anti-tumor activity [1]. Inhibition of this immune checkpoint (IC)-control mechanism by blocking PD-L1–PD-1 interaction “normalizes” the immune response with relatively low immune-related adverse events [2, 3]. For patients with mismatch repair deficient stage II or III rectal cancer, PD-1 antibody (dostarlimab) monotherapy exerted a complete clinical response [4], underscoring the remarkable potential of immune checkpoint inhibitors for certain types/subtypes of cancer. For TNBC patients with PD-L1 positive tumors, a phase 3 clinical trial revealed a median overall survival of 27 months following combination treatment with the anti-PD-L1 monoclonal antibody, atezolizumab, plus nab-paclitaxel compared with 15.5 months for nab-paclitaxel-only therapy [5, 6]. However, initial results from IMpassion131 revealed no improvement in progression or overall survival in atezolizumab with paclitaxel versus paclitaxel alone, highlighting the importance of patient selection and/or the need for targeting additional IC modulators for successful therapy of TNBC [6, 7].

PD-L1 is a 290-amino acid transmembrane protein with a calculated molecular weight of 33 kDa. N-glycosylation increases its stability and ligand binding and reduces its mobility to an apparent molecular weight of 40–55 kDa [8]. Cleavage, splice variants and other forms with unknown functions have also been documented [9–11]. PD-L1 expression and activity are regulated by multiple oncogenic pathways, including MYC and NF-kB [12, 13].

The poliovirus receptor (PVR, CD155) inhibits natural killer cells (NK) by binding T-cell immunoreceptors with Ig and ITIM domains (TIGIT) or CD96 expressed on NK cells to modulate innate immune response [14]. Its expression is associated with poor response to anti-PD-1/PD-L1 therapy [15–17]. PVR, a glycosylated transmembrane protein involved in cell proliferation, adhesion and motility/metastasis, is upregulated in many cancers [18–20].

The retinoblastoma tumor suppressor gene, *RB1*, is often disrupted together with *TP53* in TNBC and in metastatic BC [21–23]. Its product, pRB, regulates cell-cycle progression, survival, and differentiation by binding to and regulating transcription factors such as members of the E2F family. These transcription factors include three subclasses: canonical activators (E2F1, E2F2, E2F3a/b), canonical repressors (E2F4-6), and atypical repressors (E2F7-8) ([24] and references therein). pRB binds activator E2F1-3 during most of the G1 phase of the cell cycle; its sequential phosphorylation via cyclin-dependent kinases, CDK4/6 and CDK2, in response to mitogens disrupts its interaction with E2Fs, leading to cell cycle progression [25]. In TNBCs, *RB1* is frequently disrupted by mutations/deletion/silencing, whereas in luminal and most HER2+ BC, pRB is often inactivated by hyper-phosphorylation, rendering these tumors responsive to CDK4/6 inhibitors such as palbociclib, abemaciclib and ribociclib, which induce dephosphorylation and activation of pRB [26, 27].

pRB has been implicated in the control of PD-L1 via NF-kB in prostate cancer cells, whereby Ser249/Thr252-phosphorylated pRB binds and sequesters NF-kB (p65), preventing it from transcriptionally activating *CD274* [28]. In addition, CDK4 was shown to phosphorylate SPOP, stimulating the E3 ligase cullin3^SPOP^ and leading to proteasomal degradation of PD-L1 in human TNBC cells. The CDK4/6 inhibitor, palbociclib, was reported to induce PD-L1 expression in cancer cells, including TNBC [28–30]. These results raise the question of whether CDK4/6-mediated phosphorylation and inactivation of pRB also suppress PD-L1 expression, a possibility that is inconsistent with observations that multiple oncogenic alterations such as MYC, RAS, PIK3CA activation, and NF1 and TP53-loss induce PD-L1 to promote immune-evasion and cancer progression [31]. Whether pRB directly induces or suppresses the expression of PD-L1 and other IC modulators is of paramount importance for understanding how this tumor suppressor promotes TNBC progression. Here we show that both CDK4/6 inhibitors and pRb suppress PVR and PD-L1, whereas RB knockdown and E2F1 overexpression induce these checkpoint proteins. We further show that previous reports on the induction of PD-L1 by palbociclib is likely due to HCl, used to solubilize palbociclib, which acts as a strong inducer of this IC modulator. Furthermore, lactic acid, a by-product of glycolysis, induces both PD-L1 and PVR, underscoring a mechanism by which physiological acidification can suppress immune surveillance. Ramifications of these results for cancer progression, anti-CDK4/6- and IC-therapies are discussed.

## Results

### *Rb*-loss in a mouse model of TNBC reduces hallmarks of immune response

**Fig. 1:**
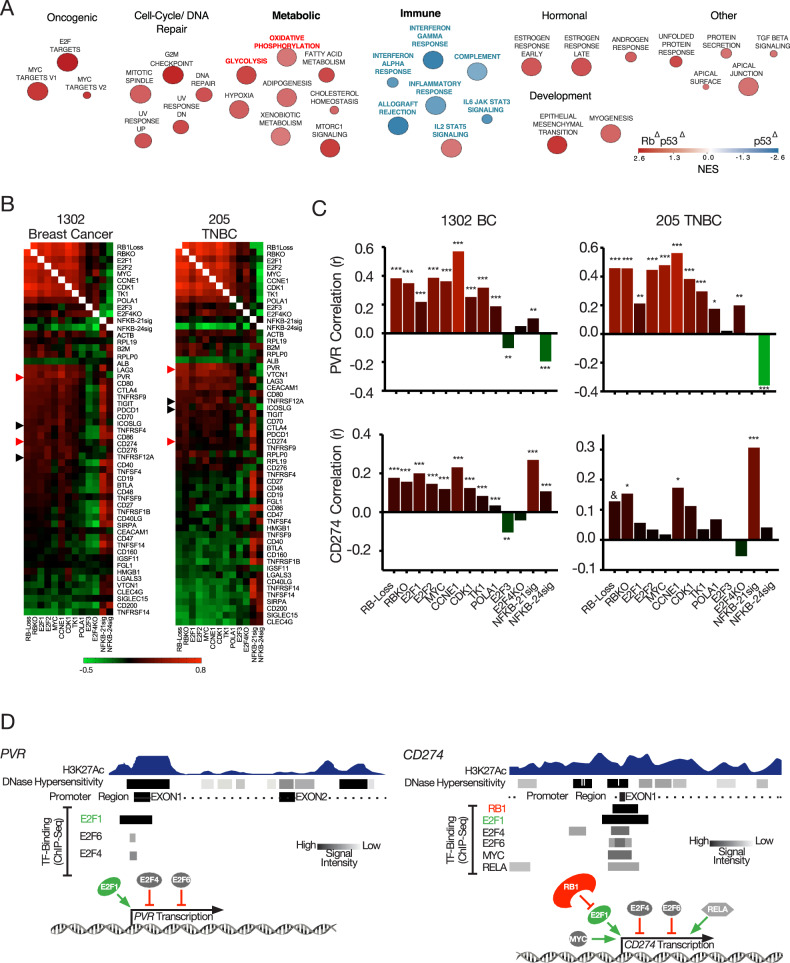
RB-loss correlates with reduced expression of immune hallmarks and increased RNA expression of *CD274* and *PVR* in mouse and human breast cancer. **A** GSEA comparing immune-related pathways in Rb^Δ^p53^Δ^ (red) with p53^Δ^ (blue) TNBC-like mammary tumors, visualized by Cytoscape. NES normalized enrichment score. **B** Correlation analysis of immune markers (right labels) with molecular signatures (bottom labels) in patient samples of 1302 mixed breast cancer and 205 TNBC. **C** Correlation level of *CD274* (PD-L1) and *PVR* with indicated RB-related pathways. **D** Summary of ENCODE analysis for the *PVR* and *CD274* promoter region (see Supplementary Fig. S3A). *P < 0.05, **P < 0.001, ***P < 0.0001, ^&^*P* = 0.0659.

We previously demonstrated by Gene Set Enrichment Analysis (GSEA) that immune response pathways are downregulated in *Rb:p53*-deficient vs. *p53*-deficient murine models of TNBC [23]. To further probe the effect of *Rb*-loss on the immune microenvironment in these isogenic tumors, we analyzed the data using Hallmark Molecular Signatures (Fig. 1A, Supplementary Fig. S1). Relative to *p53*-loss alone, *Rb/p53*-deficient TNBC-like tumors exhibited reduced interferon alpha and gamma, complement, inflammatory response, allograft rejection and IL6-JAK-STAT3 signatures. *Rb/p53*-deficient tumors also showed enrichment in oncogenic hallmarks such as E2F and MYC targets, cell-cycle/DNA repair, oxidative phosphorylation, glycolysis, fatty acid metabolism, adipogenesis as well as epithelial-mesenchymal transition and myogenesis. Thus, *Rb*-loss enhances cancer by inducing hallmarks of tumorigenesis and by suppressing hallmarks of immune response. This analysis also reveals that *Rb* loss induces both OXPHOS- and glycolytic- pathways, suggesting intra-tumoral heterogeneity or a hybrid metabolic state (see “Discussion”).

### Expression of PD-L1, PVR and other immunomodulators correlates with RB-loss and E2F1/2 expression in human breast cancer

To identify immunomodulatory genes regulated by pRB, we performed a correlation analysis between RB-loss signatures and related pathways versus RNA expression of multiple immunomodulators. We analyzed 1302 BC samples of all subtypes as well as a cohort of 205 TNBC samples using RB knockout (RBKO), RB-loss, E2F1-3 and E2F4KO signatures, and cyclin D1, cyclin E1 and MYC expression, as described [32–34] (Fig. 1B, C, Supplementary Fig. S2A–C). For NF-κB, we employed two signatures: NF-κB-activated recurrence predictor (21 genes) and matched human homolog genes from NF-κB-activated intact (no castration) mouse prostate (24 genes) [35]. In both mixed BC and TNBC subtypes, the natural killer (NK) cell adhesion checkpoint modulator, poliovirus receptor (*PVR*) gene, scored the highest correlation with RBKO, while *LAG3*, *CTLA4*, *TIGIT*, *ICOSLG*, *CD70*, *CD274 (PD-L1)*, *CD276* and *PDCD1* consistently correlated with RBKO, E2F1, E2F2 signatures and MYC expression as well as with the E2F1 targets: *CCNE1, CDK1, TK1* and *POLA1*. *PVR* correlated with the RBKO signature with *r* = 0.3488 (*P* < 0.0001) and *r* = 0.4568 (*P* < 0.0001) in mixed and TNBC BC subtypes, respectively. *PVR* consistently scored higher correlations with TNBC than with mixed BC, despite a much smaller sample size, indicating specificity to TNBC. RB-loss and RBKO signatures had a correlation of 0.177 (*P* < 0.0001) and 0.157 (*P* < 0.0001) with *CD274* in mixed BC and a correlation of 0.128 (*P* = 0.0668) and 0.154 (*P* = 0.028) in TNBC, respectively. For comparison, *STAT3* (BC: *r* = 0.18420, *P* < 0.0001; TNBC: *r* = 0.18274, *P* = 0.009) and *MYC* (BC: *r* = 0.119, *P* < 0.0001; TNBC: *r* = 0.018, *P* = 0.789), both known transcriptional activators of *CD274* [12, 36], showed similar or lower correlations (Fig. 1B, C; Supplementary Fig. S2A–C). The correlation levels of RB-loss and high E2F signatures with *PVR* (*r* = 0.21–0.48) and *PD-L1* (*r* = 0.15–0.20) are a bit lower but in the same range as bona fide E2F1 regulated cell cycle genes such as *TK1* (*r* = 0.46–0.67), *CDK1* (*r* = 0.62–0.88) and *POLA1* (*r* = 0.22–0.45) (Supplementary Fig. S2B, C). We focused our attention on PVR and PD-L1, but the effect of pRB on the other immunomodulators likely contributes to its overall impact on immune surveillance.

### Chromatin immunoprecipitation-sequencing (ChIP-seq) analysis via ENCODE reveals direct recruitment of pRB and E2F1/4/6 to the promoters of *PVR, CD274* (*PD-L1*), *ICOSLG* and *TNFRSF12A*

To ask whether pRB and activating E2Fs directly regulate the expression of immunoregulatory genes, we searched the Encyclopedia of DNA Elements (ENCODE) database (https://www.encodeproject.org)(consortium, 2012) for pRB-E2F recruitment to promoters of genes listed in Fig. 1B (between *PVR* and *CD274*). Based on chromatin immunoprecipitation sequencing (ChIP-seq) analysis of multiple cell lines, the *PVR*, *CD274*, *ICOSLG* (Inducible T Cell Co-Stimulator Ligand) and *TNFRSF12* *A* (TNF Receptor Superfamily Member 12A) promoters recruit E2F1 (Fig. 1D; Supplementary Fig. S3A). Specifically, the *PVR* promoter contains DNase1 hypersensitive (DHS) region and H3K27-acetylation marks, indicative of actively transcribed genes, that also recruit E2F4 and E2F6, with E2F1 showing the strongest intensity (Fig. 1D, Supplementary Fig. S3A). In addition, ENCODE ChIP-seq analysis with HA antibody of the luminal BC cell line MCF7 transduced with HA-E2F1 revealed peaks within this region.

The *CD274* promoter contains two major DHS regions, I and II, with the latter overlapping the transcription start site (Fig. 1D). Peaks of H3K27-acetylation are found between DHS-I and II as well as downstream regions. ENCODE ChIP-seq data revealed the recruitment of pRB, E2F1, E2F4 and E2F6, as well as MYC and RELA to the DHS-II region. Another E2F4 binding site is located around DHS-I. As expected, STAT3 is also recruited to the *CD274* promoter (Supplementary Fig. S3A). Finally, ChIP-seq analysis of MCF7 cells transduced with HA-tagged E2F1 revealed its occupancy overlaps DHS-II (Fig. 1D; Supplementary Fig. S3A).

Importantly, E2F1 affinity peaks at the *PVR* and *CD274* promotors are similar in their intensity to those seen in bona fide E2F-regulated targets (*CCNE1*, *CDK1*, *DHFR*, *RBL1*, *RB1*; Supplementary Fig. S3A and C), supporting the idea that these immunomodulatory genes are genuine E2F1 targets. Other immunomodulatory genes whose expression correlates with high RB-loss and E2F1/2 signatures, such as *CD80*, *CTLA4*, *LAG3*, *TIGIT* but exhibited no or very little RB-E2F recruitment to their promoters in MCF7 cells (Supplementary Fig. S3B), may represent indirect consequences of RB-E2F dysregulation. As a negative control, the albumin gene *ALB* also showed no RB-E2F recruitment (Supplementary Fig. S3B). Interestingly, our ENCODE analysis revealed that *STT3A* and *STT3B*, which induce PD-L1 glycosylation [37], also recruit pRB and activating-E2Fs to their promoters (Supplementary Fig. S3D), suggesting multiple levels of regulation of PD-L1 expression by the pRB-E2F tumor suppressor axis.

### The pRB-E2F axis controls PVR expression

**Fig. 2:**
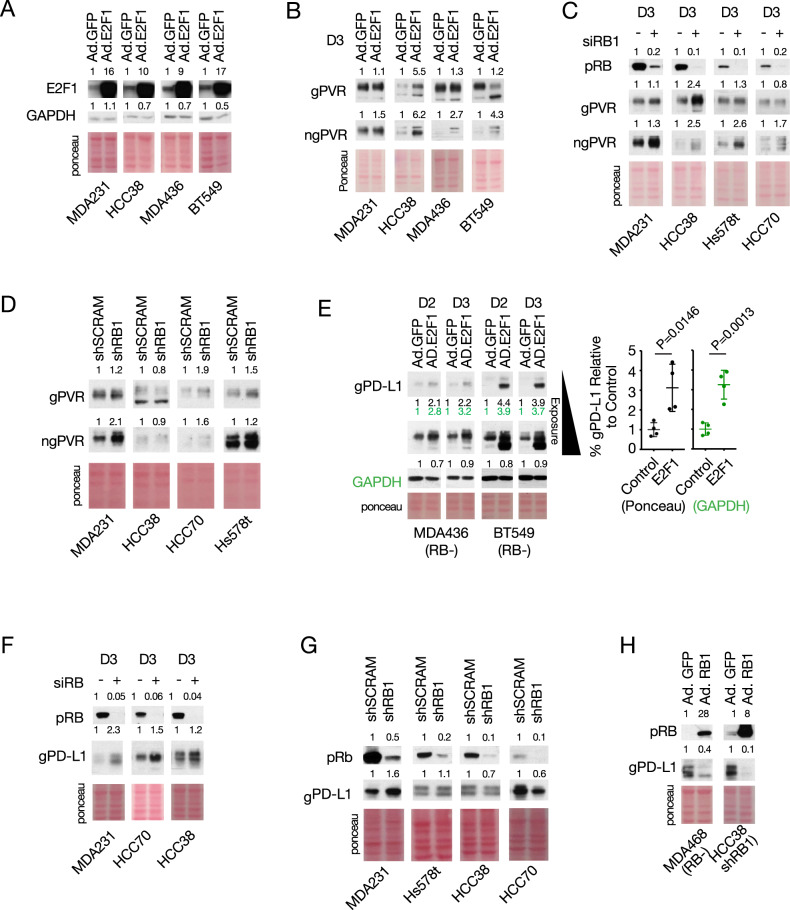
Direct Regulation of PVR and PD-L1 by pRB and E2F1. **A** Immunoblot validation of E2F1 expression following adenovirus-mediated transduction of *E2F1* (Ad.E2F1) in indicated TNBC lines. **B** Immunoblot analysis of glycosylated and non-glycosylated PVR in multiple TNBC lines transduced with Ad.GFP or Ad.E2F1, or **C** depleted for RB1 via RNAi. **D** Immunoblot analysis of PVR response to stable depletion of RB1 by shRNA. **E** Left, immunoblot analysis of PD-L1 in RB1-deficient TNBC lines transduced with Ad.GFP or Ad.E2F1. Right, cumulative PD-L1 quantification normalized to ponceau (black) or to GAPDH (green). **F** Immunoblot analysis of PD-L1 in three different RB(+) TNBC lines following acute knockdown of RB1 via RNAi, 3 days (D3) post transfection. **G** Immunoblot analysis of PD-L1 in TNBC lines with stable *RB1* knockdown. **H** Immunoblot analysis of PD-L1 in *RB1*-deficient (MDA-MB-468) or *RB1*-depleted (HCC38-shRNA) TNBC cell lines transduced with Ad.RB1. Numbers above immunoblots denote band intensity normalized to loading control.

To specifically probe the effect of pRb and E2F1 on PVR protein expression, we first transduced cancer cells with a recombinant adenovirus encoding E2F1 (Ad.E2F1). Overexpression of E2F1, confirmed by immunoblots (Fig. 2A), robustly induced non-glycosylated (ngPVR) and/or glycosylated (gPVR) PVR (Fig. 2B). Acute RB knockdown via RNAi also induced ngPVR in all lines tested (Fig. 2C). Finally, we generated four isogenic cell lines in which endogenous *RB1* expression was stably knocked down via lenti-RB1^shRNA^. Similar to the effect of transient depletion, stable knockdown of *RB1* induced ngPVR and gPVR in all lines, with the exception of HCC38 (Fig. 2D).

### The pRB-E2F axis controls PD-L1 expression

To ask whether the RB-E2F1 axis also regulates PD-L1 expression, we transduced TNBC cells with Ad.E2F1 or transiently knocked-down RB via RNAi. Western blot analysis of the RB-proficient BC lines MCF7, MDA-MB-231 and HCC38, RB-shRNA knockdown lines (MDA-MB-231, HCC38) or RB-deficient lines MDA-MB-436 and BT549 revealed that E2F1 overexpression significantly induced non-glycosylated (ngPD-L1) and glycosylated (gPD-L1) PD-L1 in all lines compared to controls (Fig. 2E, Supplementary Fig. S4A–C). Similar results were observed when we concurrently transduced the cells with an adenovirus encoding for the survival factor Bcl-2 to counteract apoptosis induced by E2F1, as we previously described [23] (Supplementary Fig. S4A–C). Quantification of the combined levels of each PD-L1 form revealed that both gPD-L1 and ngPD-L1 were significantly upregulated (Fig. 2E, Supplementary Fig. S4A–C). Notably, normalization for GAPDH and ponceau loading controls showed similar induction levels of PD-L1 (Fig. 2E, Supplementary Fig. S4C). As ponceau validates transfers across the whole lane and its expression does not vary by experimental conditions as it may with single “house-keeping” proteins, we proceeded to use it as a loading control in all subsequent experiments.

Transient depletion of *RB1* via RNAi also induced gPD-L1 in all three RB-proficient TNBC lines tested: MDA-MB-231, HCC38 and HCC70 (Fig. 2F). Finally, we determined the effect of stable depletion of *RB1* on PD-L1 expression. Compared to controls (shSCRAM), shRB1-depletion induced gPD-L1 expression in MDA-MB-231 and, to a lesser extent, in Hs578t cells (Fig. 2G). In contrast, stable knockdown of *RB1* in HCC38 and HCC70 cells, in which transient inactivation of *RB1* via RNAi enhanced gPD-L1 expression (Fig. 2F), led to reduced levels of this immunomodulator (Fig. 2G), demonstrating again, context-specific effects.

Next, we asked whether transduction of *RB1* via an adenovirus vector (Ad.RB1) would exert the opposite effect seen following RB-loss or E2F1 overexpression on PD-L1 expression. Ad.RB1 infection strongly suppressed gPD-L1 in RB-deficient MDA-MB-468 cells and in RB1-depleted (via shRB1) HCC38 cells (Fig. 2H). These results suggest the RB-E2F1 pathway regulates both PVR and PD-L1 with some cell-specific effects of stable vs. transient depletion of *RB1*.

### The CDK4/6 inhibitor palbociclib suppresses PVR and PD-L1 expression

**Fig. 3:**
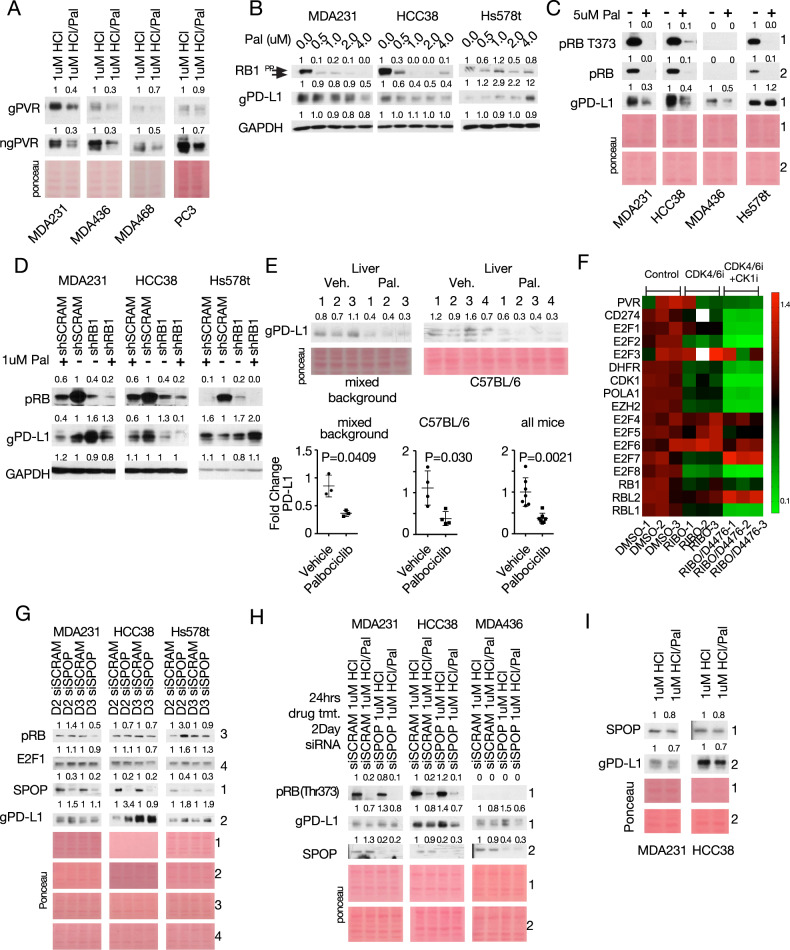
The CDK4/6 inhibitor palbociclib suppresses PVR and PD-L1 expression, and counteracts the effect of SPOP depletion on PD-L1. **A** Immunoblot analysis showing the effect of 2-day palbociclib treatment on ngPVR and gPVR expression. **B**, **C** Immunoblot analysis of PD-L1 in TNBC lines treated with increasing (**B**) or high (**C**) concentrations of palbociclib for 2 days. **D** Immunoblot analysis of PD-L1 in TNBC lines with stable knockdown of *RB1*, treated with/without palbociclib for 2 days. **E** Immunoblot analysis of PD-L1 in livers of mice on mixed background treated with palbociclib (140 mg/kg; gavage; 5 consecutive days/week) for 28 days and probed with AF1019 antibody (left) or in livers of C57BL/6 mice treated with palbociclib (140 mg/kg; gavage) for 7 consecutive days, probed with MAB90781 antibody (right). Bottom, PD-L1 quantification. Pure palbociclib (FA65120) was suspended in sodium lactate (50 nM, PH 4.0), which was used in the vehicle control mice. **F** Heatmap analysis of RNA-seq data (GSE177054) of MDA231 treated with ribociclib or ribociclib/D4476 for 2 days. White boxes denote out-of-range intensity. **G** Immunoblot analysis of PD-L1 in indicated tumor cells following knockdown of SPOP by RNAi over 2 (D2) or 3 (D3) days. **H** Immunoblot analysis of PD-L1 in indicated TNBC lines with/without SPOP knockdown after 2 days (D2) and treated with/without palbociclib for 24 h. **I** Immunoblot analysis of PD-L1 and SPOP in indicated TNBC lines after palbociclib treatment for 48 h. Numbers above immunoblots denote band intensity normalized to loading control.

Our observation that pRB represses PVR and PD-L1 expression via E2F1 suggested that CDK4/6 inhibitors, such as palbociclib, which block pRB phosphorylation and its dissociation from E2F1, would also diminish the expression of these IC proteins. Palbociclib exerted cell-specific effects on gPVR and ngPVR, with dramatic inhibition of both forms in MDA-MB-231, MDA-MB-436 and MDA-MB-468 TNBC as well as PC3 prostate cells, a slight reduction in BT549 TNBC cells, but induction in HCC38 TNBC cells (Fig. 3A, Supplementary Fig. S5A).

Palbociclib also reduced expression of gPD-L1 in MDA-MB-231 and HCC38 cells dose-dependently (0.5–4 µM), but induced it in Hs578t cells (Fig. 3B). Similar results were observed even at a higher concentration of 5 µM (Fig. 3C). Palbociclib blocked pRB phosphorylation on Serine 373 in all these lines indicating its differential effect on PD-L1 expression is not due to its inability to block pRB phosphorylation in Hs578t cells (Fig. 3C). In MDA-MB-231 cells, stable knockdown of *RB1* via shRNA increased gPD-L1 while palbociclib treatment decreased it (Fig. 3D). In HCC38, *RB1* knockdown decreased gPD-L1, whereas palbociclib treatment reduced gPD-L1 in both parental and RB-depleted cells (Fig. 3D). In contrast, in Hs578t cells, in which *RB1* knockdown increased PD-L1 expression, palbociclib treatment further induced its expression, demonstrating cell-specific effects of combined CDK4/6 inhibitors plus stable RB depletion on PD-L1 expression (Fig. 3D). Palbociclib also suppressed gPD-L1 in RB-proficient HCC3153 and RB-negative MDA-MB-436, BT549 TNBC cell lines, as well as in the prostate cancer line PC3 (Supplementary Fig. S5B). In contrast, palbociclib had a minimal effect on gPD-L1 in the TNBC cell line, MDA-MB-468, and the trastuzumab-resistant HER2+ breast cancer cell line, JIMT1, whereas in the HER2-enriched cell line, HCC1954, gPD-L1 was slightly induced (Supplementary Fig. S5B). These results emphasize again, like in the case of PVR, that palbociclib suppresses these IC modulators in most, though not all, cell lines, exposing the cell-specificity of their regulation and response to CDK4/6 inhibition.

To ask whether palbociclib also suppresses gPD-L1 expression in vivo, we treated mice by gavage for 4 weeks (5 days/week) or for only 7 straight days with palbociclib (140 mg/kg), dissolved/suspended in sodium lactate (50 nM, pH 4.0). As the majority of palbociclib is metabolized in the liver (https://pubchem.ncbi.nlm.nih.gov/compound/Palbociclib,www.pfizermedicalinformation.com/en-us/ibrance/clinicalpharmacology), we specifically analyzed its effect on this tissue. In agreement with the in vitro analysis, liver tissues from palbociclib-treated mice exhibited significantly reduced gPD-L1 expression compared to vehicle-treated mice, and this was observed using two different PD-L1 antibodies (Fig. 3E).

To further interrogate the effect of CDK4/6 inhibition on *CD274*, we analyzed published RNA-seq data of MDA-MB-231 cells treated with another CDK4/6 inhibitor, ribociclib or ribociclib plus D4476 (a protein kinase CK1 inhibitor that stabilizes pRB) (GSE177054) [38]. Ribociclib treatment alone decreased and further synergized with CK1 inhibition to markedly diminish *CD274* and *PVR* expression, along with bona fide E2F-regulated genes: *E2F1/2*, *DHFR*, *CDK1*, *POLA1*, *EZH2* and increased E2F-target inhibitors: *E2F4/5/6/7* (Fig. 3F). Thus, both palbociclib (this study) and ribociclib (ibid) suppress *PD-L1* and *PVR* gene expression.

### Palbociclib counteracts the effect of SPOP depletion on PD-L1 expression

CDK4-mediated phosphorylation of SPOP was shown to induce the E3 ligase Cullin3^SPOP^, which degrades PD-L1 [29]. We therefore determined the effect of SPOP depletion via RNAi on gPD-L1 expression alone or together with palbociclib. In three different TNBC lines, exposure to siSPOP led to a reduction in SPOP protein level and induction of gPD-L1 relative to control siSCRAM (Fig. 3G). Next, cells were treated with siRNAs, trypsinized the next day, reseeded and exposed to palbociclib or vehicle alone so that treatments were performed on the same cell pools. While siSPOP increased gPD-L1 expression, palbociclib suppressed gPD-L1 in both siSPOP- and siSCRAM-treated cells compared to vehicle-treated cells in the pRB+ MDA-MB-231 and HCC38 cells as well as in the RB-deficient TNBC line MDA-MB-436 (Fig. 3H, Supplementary Fig. S6A, B). As CDK4-mediated phosphorylation of SPOP stabilizes it, we expect palbociclib to block CDK4 and reduce the level of SPOP. This is seen in two of three lines in Fig. 3H after a short exposure of 24 h to Palbociclib (in the absence of SPOPi). We repeated the experiment with a longer exposure (48 h) to better reveal changes in protein stability/degradation. Under these conditions, palbociclib exhibited a small (20%) but consistent inhibition of SPOP expression level (Fig. 3I). In these experiments, palbociclib reduced SPOP level, yet it also reduced overall gPD-L1 expression, suggesting a model in which CDK4 controls PD-L1 turnover by promoting PD-L1 degradation via SPOP, while concomitantly inducing PD-L1 transcription via E2F1 by phosphorylating and inhibiting pRB (see “Discussion”).

**Fig. 4:**
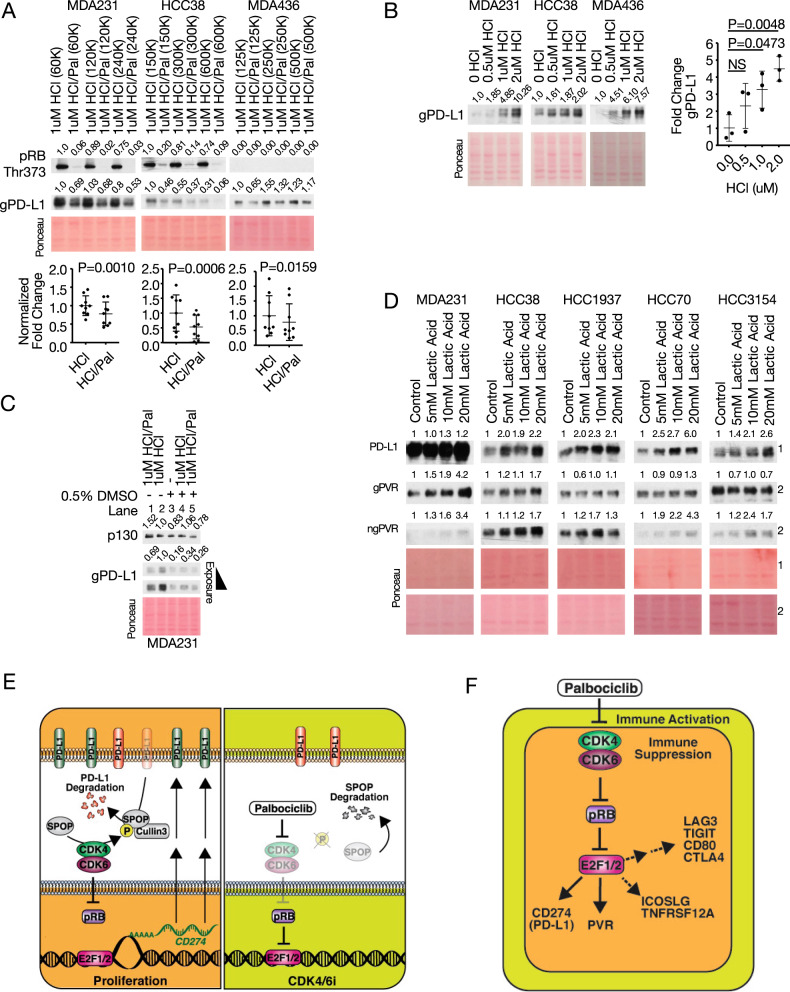
Effect of confluency, palbociclib treatment duration, hydrochloric and lactic acids on PVR and PD-L1 expression in TNBC cells. **A** Immunoblot analysis of PD-L1 in TNBC lines seeded at various densities in 6-cm plates and treated with 1 µM palbociclib for ~2 days. Bottom, PD-L1 quantification of (**A**) and Supplementary Fig. 7A. Statistics calculated by pairwise analysis of seeding densities and treatment durations. **B** Immunoblot analysis of TNBC lines treated with various concentrations of hydrochloric acid (HCl) for 2 days. Right, PD-L1 quantification and analysis by Welch’s *t*-test. **C** Immunoblot analysis of MDA231 treated with/without palbociclib and/or with/without indicated solvents. **D** Immunoblot analysis of PD-L1 and PVR in different TNBC lines treated with physiological concentrations of lactic acid for 2 days. Numbers above immunoblots denote band intensity normalized to loading control. **E** A model suggesting that in response to mitogenic signals, CDK4/6 controls PD-L1 turnover by inducing its degradation via Culin3^SPOP^ and its transcription via pRB-E2F1. PD-L1 in red and green depicts old or newly synthesized glycosylated protein, respectively. **F** A model suggesting that the CDK4/6-RB-E2F axis modulates immune surveillance by transcriptionally regulating PD-L1, PVR and potentially other IC modulator genes. Solid and segmented arrows indicate validated (herein) and unvalidated targets, respectively; single arrows denote pRB and E2F1 binding sites as well as strong E2F1 recruitment by ChIP-seq analysis, whereas double arrows denote no or weak E2F1 recruitment.

The above results, whereby palbociclib suppresses gPD-L1 expression in multiple TNBC cell lines, are in sharp contrast with previous reports showing induction of gPD-L1 by this CDK4/6 inhibitor [28–30]. To rectify this discrepancy, we first asked whether palbociclib treatment duration or confluency through contact inhibition might dampen transcriptional responses in favor of SPOP-degradation and increased PD-L1 stability. MDA231, HCC38 and MDA436 cells were seeded at three different densities so that at the highest density, cells were ~80% confluent after 48–50 h or fully confluent after 60 h in the vehicle-treated arms. Palbociclib reduced gPD-L1 in all lines, at all densities and treatment durations (Fig. 4A, Supplementary Fig. S7A). Notably, in the absence of palbociclib, gPD-L1 expression decreased in the RB-proficient cell lines (MDA-MB-231, HCC38) with increasing confluency but not in the RB-deficient MDA-MB-436 cell line. We also examined if the effect of various lysis conditions impacted PD-L1 expression. Both ionic (RIPA; Na deoxycholate) and non-ionic (NP-40; TritonX 100) lysis buffers consistently showed that RB-loss increased gPD-L1 in shRB vs. shSCRAM control MDA-MB-231 cells, whereas palbociclib decreased gPD-L1 in all samples regardless of the lysis buffer (Supplementary Fig. S7B, C).

### Hydrochloric acid, used to solubilize palbociclib, induces PD-L1 expression

We noticed that all previous reports in which palbociclib induced rather than suppressed PD-L1 employed acidified palbociclib and DMSO. Pure palbociclib is highly insoluble in water and DMSO but is readily solubilized under acidic conditions. “Acidified” palbociclib is readily soluble in water, negating solubilization in DMSO and avoiding its potential confounding effects. For experimental use in vitro, palbociclib is commercially available premixed with HCl or isethionate (1:1 ratio, not chemically bound) in equal molarity to increase solubility. In all experiments described so far, we used pure palbociclib (not palbociclib:HCl), which we dissolved in an equimolar HCl solution that was also added to the vehicle-alone control. Importantly, treatment with HCl alone or together with DMSO robustly induced PD-L1 expression. Thus, when TNBC cells were exposed to increasing doses of HCl (without DMSO), HCl concentrations of 0.5–2 µM induced gPD-L1 dose-dependently in all three tested lines (Fig. 4B). The effect of HCl was stronger in non-confluent than in confluent cultures but was clearly seen under both conditions (Supplementary Fig. S8). We then compared the effect of DMSO, which is often used as a control vehicle, on PD-L1 expression, alone or together with HCl (Fig. 4C). When palbociclib was solubilized in HCl and compared to control DMSO alone (lanes 1 vs. 3), it induced gPD-L1. In contrast, when properly controlled, palbociclib suppressed gPD-L1 expression both in the HCl alone (lanes 1 vs. 2) and in the HCl plus DMSO arms (lanes 4 vs. 5). Thus, HCl induces while DMSO reduces PD-L1.

We next solubilized commercially available Palbociclib:HCl in DMSO or water. Treatment of DMSO/Palbociclib:HCl vs. DMSO showed no discernible effect on gPD-L1 expression in three different TNBC lines (Supplementary Fig. S9, left). However, when palbociclib:HCl (without DMSO) was compared to DMSO alone, it substantially increased gPD-L1 in all three lines (Supplementary Fig. S9, right). The effect of HCl was specific to gPD-L1 as p107, pRB, E2F1 and E2F4 protein levels were not altered in these uncontrolled sets, whereas p130 was slightly altered (Supplementary Fig. S9). Furthermore, these cell cycle regulators showed expected responses to palbociclib in both uncontrolled sets; dephosphorylation of all three pocket proteins, reduction of activator E2F1 and induction of inhibitor E2F4 (Supplementary Fig. S9). These results demonstrate that the impact of HCl versus vehicle conditions is specifically critical for gPD-L1, is antagonistic to palbociclib, and must therefore be carefully controlled in the vehicle-alone arm when analyzing its response to palbociclib.

### Lactic acid also induces PD-L1 and PVR expression

Our observations that HCl induces PD-L1 and that RB-loss induces glycolysis prompted us to ask whether lactic acid, the secreted by-product of glycolysis, also affects PD-L1 expression. Tumor-relevant concentrations of lactic acid were added to cultured cells at the indicated concentration (5–20 mM) [39] for 48 h, followed by immunoblotting for the IC modulators. Exposure to lactic acid induced gPD-L1 in all lines tested and the glycosylated and non-glycosylated forms of PVR (gPVR; ngPVR) in multiple TNBC lines (Fig. 4D). Thus, the induction of PD-L1 by HCl is not an extraneous laboratory artifact but a reflection of a physiologically important effect of acidity on this checkpoint protein as well as on PVR. Moreover, these results suggest that RB-loss not only induces IC modulators at the transcriptional level, like multiple other oncogenic signaling [31] but also through lactic acid secretion via glycolysis which enhances the level of these proteins (Figs. 1A and 4D).

## Discussion

We report direct transcriptional regulation of the NK regulator *PVR* and the T-cell modulator *CD274* (PD-L1 gene) by the CDK4/6-RB-E2F axis. Rb-loss reduces several ‘hallmarks’ of immune response in mouse models, and its deficiency correlates with the induction of *PVR, CD274* and several other IC modulators, including *ICOSLG* and *TNFRSF12A* in human breast cancer. Here, we show that E2F1 induction and RB depletion induce PVR and PD-L1 protein expression in TNBC cells.

Overexpression of E2F1 and acute RB depletion via RNAi invariably induced PVR and PD-L1. However, chronic depletion of *RB1* in TNBC cells via stable, lenti-shRNA infection led to induction of gPD-L1 in two lines but suppression in two other (in which induction was observed after transient RB depletion), indicating possible cell-specific compensation by other pocket proteins [40] or other factors. Overexpression of E2F1 likely overcomes such compensatory mechanisms, leading to the induction of PVR and PD-L1 in all tested cells. We also show that E2Fs and pRB are recruited to the *STT3* promoter, suggesting that the pRB/E2F axis coordinates PD-L1 gene transcription with its glycosylation, a possibility that warrants further investigation.

Consistent with our observation that pRB suppresses *PVR* and *PD-L1*, we demonstrated that the CDK4/6 inhibitor palbociclib diminished the expression of these immunomodulators. Moreover, palbociclib counteracted the effect of SPOP depletion, which stabilizes and induces PD-L1 expression [29]. Palbociclib also suppressed gPD-L1 in liver tissues in vivo. In addition, another CDK4/6 inhibitor, ribociclib, in combination with CK1 inhibition (which stabilizes pRB), reduces both *CD274* and *PVR* mRNA expression in a published dataset.

We show that HCl, often used to solubilize palbociclib, induces PD-L1 expression, counteracting the effect of palbociclib, and therefore must be properly controlled in the vehicle-alone arm. This may explain previous reports showing that palbociclib induces gPD-L1 expression in TNBC, prostate and melanoma cell lines [28–30].

Thus, while CDK4/6 phosphorylation of SPOP induces PD-L1 degradation through the Cullin3^SPOP^ E3 ligase [29], its phosphorylation of pRB, dissociates this tumor suppressor from activating-E2Fs, leading to transcriptional activation of *CD274* (PD-L1) and the generation of new PD-L1 proteins, with a net increase in PD-L1 level. CDK4/6 may thus function to control PD-L1 turnover by coupling degradation of “old” PD-L1 with transcription and subsequent synthesis of “new” PD-L1 proteins (Fig. 4E). In response to mitogenic signals, newly generated daughter cells must produce PD-L1 to maintain high expression of this IC protein to evade immune-destruction. The need to degrade existing (old) PD-L1 is not obvious, though it may increase plasticity by allowing the replacement of different forms of PD-L1 on the cell surface. Additional analysis is needed to demonstrate the actual turnover of PD-L1 in response to mitogens through CDK4/6 and elucidate the immunological importance of this process.

The CDK4/6-pRB-E2F axis regulates not only NK cells (via PVR) and cytotoxic T cells (via PD-L1) but multiple other immune cells; type-2 T helper cells (ICOSLG), and B cells, CD4^+^ T cells, CD8^+^ T cells, neutrophils, macrophages, and dendritic cells (TNFRSF12A). Thus, this axis acts to suppress both innate and adaptive immune responses, allowing newly generated daughter cells to thrive during normal homeostasis as well as cancer invasion (Fig. 4F). Conversely, CDK4/6 inhibitors such as palbociclib shuts down E2F1/2-mediated transcription of these IC modulators, leading to immune rejection. This model is consistent with the observation that depletion of pRB in MCF7 cells increases tumorigenesis upon transplantation into SCID mice [41]; however, additional experiments are required to assess the importance of RB regulation of IC modulators on tumor progression in RB-deficient cancers. As noted, despite the initial promising results from PD-L1-based clinical trials of advanced TNBC, the effect of this therapy is still controversial. Our results suggest a testable prediction that IC inhibitors for PVR, ICOSLG or TNFRSF12A, either alone or in combination with anti-PD-L1, may unleash a more effective immune response against TNBC. Notably, poor response to anti-PD-L1–PD-1 therapies is associated with PVR expression. Multiple clinical trials are underway targeting both PVR (using NTX-1088 [15, 17] or the PVR partner TIGIT) and PD-L1/PD-1 (https://clinicaltrials.gov/ct2/show/NCT05378425?term=pvr&cond=Cancer&draw=2&rank=1, https://clinicaltrials.gov/ct2/results?cond=Cancer&term=tigit+pd-l1&cntry=&state=&city=&dist=&Search=Search), and our results suggest that such efforts may be particularly effective against RB1/TP53-deficient TNBC.

Notably, our previous analysis of Rb:p53-deficient vs. p53-deficient TNBC-like mouse model revealed induction of mitochondrial protein translation (MPT) and oxidative phosphorylation (OXPHOS) [23, 42]. Our further analysis herein of the same data using Hallmark Molecular Signatures revealed induction of both OXPHOS and glycolysis in response to Rb loss in these isogenic tumors. There is growing evidence that plasticity, such as hybrid EMT (epithelial-mesenchymal transition), is a crucial characteristic of disseminating tumor cells [43]. *RB1* loss may promote a hybrid metabolic state with features of both OXPHOS/MPT and glycolysis. Multiple other oncogenic signaling promote glycolysis and metabolic reprogramming ([42] and references therein). Glycolytic cells secrete lactic acid, which is associated with tumor progression/immune suppression and poor prognosis in many cancers [39]. Here we show that lactic acid induces both PVR and PD-L1 expression. A recent report shows that in glioblastoma cells, glycolysis induces mitochondrial dissociation of hexokinase (HK2), which binds and phosphorylates IkB1, leading to its degradation and NF-kB-mediated stimulation of PD-L1 transcription [44]. We demonstrate that externally added lactic acid induces PD-L1 and PVR expression, likely through a different mechanism, yet to be elucidated.

Our observation that CDK4/6 inhibitors suppress PD-L1 is in line with reports on the synergy between such inhibitors and anti-PD-L1 [29, 45, 46] or anti-PD-1 [47, 48] therapy, as both diminish PD-L1 expression or PD-L1–PD-1 interaction through different mechanisms. In breast cancer, *RB1* is lost primarily in TNBC and most commonly in the BL1 subtype, which also exhibits high E2F1 and E2F2 expression as well as TP53 mutation, PTEN loss and high MYC, WNT and RHOA signaling [34]. Thus, RB1-deficient BL1 TNBC patients may particularly benefit from anti-PD-L1 and anti-PVR therapy. We and others have identified the CHK2/WEE1/CDC25/Aurora Kinase pathway as a therapeutic vulnerability in RB1-deficient TNBC [49, 50]. A combination of Atezolizumab (and/or anti-PVR) and WEE1 (MK-1775) or Aurora A Kinase (Alisertib) inhibitors may be highly effective in treating these patients, a conjecture that can now be tested directly in immunocompetent mouse models [23].

In summary, we show that CDK4/6 inhibitors and pRb both suppress PVR and PD-L1 expression by direct transcriptional repression of their promoters via activating E2Fs. We suggest that CDK4/6 controls PD-L1 turnover by coupling its degradation via cullin3^SPOP^ to its transcriptional induction via pRb-E2F1, pointing to RB1-deficient TNBC as a potential subgroup of patients who may benefit from anti-PVR plus anti-PD-L1 therapy. Moreover, our results reveal that the CDK4/6-pRB-E2F pathway controls not only innate and adaptive responses through PVR and PD-L1 but multiple other immunomodulators, suggesting a concerted, multifaced regulation of the immune surveillance machinery that can be exploited clinically.

## Material and methods

### GSEA and correlation analysis

Gene set enrichment analysis (DSEA) was performed using GSEA 4.1.0 (Broad Institute; www.gsea-msigdb.org) set at 1000 permutations, permutation type: gene_set, Chip platform: “Hallmarks”v7.5, enrichment statistic: weighted, Rank metric: Signal2Noise, Gene list sorting: real, Gene list order mode: descending, Max size: 500, Min Size:15. Cytoscape 3.8.2 used for visualization of data. For correlation analysis of the human breast cancer dataset, mRNA expression in the Illumina HiSeq RNAseq platform was obtained from the European Genome-Phenome Archive under accession numbers EGAD00010000434 [51]. NF-κB gene signatures were employed as reported [35]. A binary regression model was used to calculate pathway activity as described [32, 33]. Associations among gene expression, signatures, and pathway activities were determined by Pearson correlation.

### Cell lines

HEK293T, MCF7, MDA231, MDA436, MDA468, Hs578t, Du4475, JIMT1 and PC3 were maintained in DMEM (10% FBS, 1% penicillin/streptomycin (PEST)) at 37 °C and 5% CO_2_. BT474 and MDA361 were maintained in DMEM with 15% FBS. HCC38, HCC3154, HCC70, HCC1937, HCC1569, HCC1954 and BT549 were maintained in RPMI (10% FBS, 1% PEST). Cells were disassociated from plates with 0.5% trypsin (Sigma) after PBS washes. Freeze media (60% media, 30% FBS, 10% DMSO).

### Mice

All mouse experiments were performed in accordance with the Canadian Animals Care Council guide for the care and use of laboratory animals and were approved by the Toronto General Research Institute Animal Care Committee, UHN.

### Antibodies

RB1 (BD Pharmigen, cat. G3-245), Rabbit anti-human RB1 (Cell Signalling Technologies, cat. 9313), Phospho-RB (Thr373) (Thermo Fisher, cat. PA5-64767), Rabbit anti-human/mouse p130 (Cell Signalling Technologies, cat.13610), Rabbit anti-human p107 (Santa Cruz, cat. Sc-318), Rabbit anti-human PD-L1 (Cell Signalling Technologies, cat.13684), Goat anti-mouse PD-L1 (Novus albociclib, cat. AF1019), Rabbit anti-mouse PD-L1 (R&D systems, cat. MAB90781), PVR (Cell Signalling Technologies, cat.13544), mouse anti-human GAPDH (Santa Cruz, cat. Sc-365062), mouse monoclonal anti-human c-myc (9E10, Santa Cruz sc-40; a gift from Dr. Linda Penn), anti-goat IgG-HRP (R&D Systems, cat. HAF109), donkey anti-goat IgG-HRP (Santa Cruz, cat. Sc-2020), anti-rabbit IgG-HRP (Cell Signalling Technologies, cat. 7074), anti-mouse IgG-HRP (Cell Signalling Technologies, cat. 7076). Antibodies were diluted in 5% non-fat dry milk or BSA at dilutions of 1/1000–1/5000.

### Western blotting

#### Lysis buffers

NP-40 (50 mM Tris pH 7.5, 150 mM NaCl, 0.5% NP-40), TritonX (20 mM Tris pH 7.7, 150 mM NaCl, 1% TritonX 100, 1 mM EDTA, 1 mM EGTA), RIPA (50 mM Tris pH 7.5, 150 mM NaCl, 1% triton, 1% Na deoxycholate, 0.1% SDS, 5 mM EDTA), Nadeoxy (50 mM Tris pH 7.4, 150 mM NaCl, 1% triton 100, 1% Na deoxycholate), maltoside (10 mM Tris pH 7.4, 150 nM NaCl, 5mN EDTA, 25 mM sucrose, 5 mM glycerol, 1 mM triton 100, 1.5 mM n-dodecyle-B-D-maltoside (Thermo Fisher Scientific, cat. 89902), 0.5 mM Na deoxycholate). All lysis buffers were supplemented with 5 mM NaF, 0.5 mM Na_3_VO_4_, 10 mM PMSF and protease inhibitor cocktail (Sigma, cat. P8340). Western blotting: Cells were washed twice with PBS and lysed with one of the above lysis buffers and put on ice, scrapped into a microtube, and lysed further on ice, pelleted, and supernatants kept. Protein concentration was determined by Peirce 660 nm protein assay (Thermo Fisher, cat. 22660) on a nanodrop 2000. Then, 10–30 µg of protein were separated by SDS-PAGE and transferred overnight onto PVDF at 4 °C. Membranes were stained with Ponceau to ensure proper transfer and used to demonstrate loading amounts. Membranes were then washed with water or TBST (20 mM Tris pH 7.6, 137 mM NaCl, 0.1% Tween-20) until ponceau was removed and blocked with 5% non-fat dried milk in TBST for at least 1 h. Membranes were put into primary antibody solutions if they were in non-fat dried milk; otherwise, they were washed in TBST prior to primary antibody incubation at 4 °C overnight with gentle shaking. Membranes were then washed with TBST at least three times for 10 min each and incubated in secondary HRP conjugated antibody with gentle shaking at room temperature for 1 h or overnight at 4 °C. Membranes were then washed three times with TBST, treated with SuperSignal West Pico PLUS (Thermo Fisher, cat. 34580) for 90 s, exposed to film and developed on a Konica SRA-101A developer. Band intensity was quantified by Image lab (BioRAD) and normalized to loading controls.

### Recombinant adenovirus vectors

High-titer recombinant adenovirus particles expressing human genes were obtained from Vector BioLabs: Ad.GFP (cat. 1060), Ad.BCL2 (cat. 1412), Ad.E2F1 (cat. ADV-207490), Ad.RB1 (cat. 1043). Cells were treated with 8 µg/ml sterile polybrene 15 min prior to infection. An MOI of 250–500 was used to infect cells so that plates reach ~80% confluency 2 or 3 days post infection. Cells were washed five times on the following day with PBS and replenished with new media.

### shRNA and lentiviral generation

All pLKO.1 shRNA vectors were purchased through MISSION Sigma: non-target-puro shRNA control (cat. SHC016), shRB1-puro (TRCN0000288710). Prior to transfection, the medium of HEK293T cells in a 10-cm dish was replaced with 3.5 ml of PEST-free medium (enough to cover cells for 3 h). Equal molar ratios of packaging vector (pPAX), envelope vector (pMG2) and shRNA expression vector (pLKO.1) totaling ~16 µg DNA were mixed in 0.75 ml Opti-mem (31985062, Gibco) and vortexed for 1 min. In a separate tube, 48 µl of PEI (sterilized branched polyethylenimine, 1 µg/µl) was mixed in 0.75 ml Opti-mem and vortexed for 1 min. Both DNA/Opti-mem and PEI/Opti-mem were incubated at room temperature for 10 min; then, both solutions were mixed together with gentle pipetting 2–3 times and incubated at room temperature for 3 min. This solution was added dropwise onto HEK293T cells in 3.5 ml PEST-free media, rocked gently and incubated for 3 h at 37 °C. Cells were then washed with PBS to remove PEI and replenish it with 10 ml normal media. Media was collected after 3 days for virus and passed through 0.22-µM filters into new tubes. For infection, the filtered medium containing the virus was added to media of cells pretreated with 8 µg/ml sterile polybrene. Cells were selected based on antibiotic resistance or fluorescence by FACS (AriaIII-CFI), depending on the vector.

### siRNA/transfection

Human siRB1 (Dharmacon, cat. L-003296-02), siScrambled (Dharmacon, cat. D-001810-10), human siSPOP (Horizon, cat. L-017919-00). Transfection**:** Cells were seeded prior to transfection so that densities would be ~80% on the day of analysis/collection. For transfections, 25–50 nM RNAi concentrations were used. Using Lipofectamine RNAiMAX (Invitrogen, cat. 13778), transfection complexes were prepared with Opti-mem (Thermo Fisher, cat. 31985062) with gentle mixing and incubated for 20 min at room temperature, then added dropwise onto cells already in medium, shaken gently and incubated overnight at 37 °C. Fresh medium was replenished the next day.

### Palbociclib, HCl and lactic acid treatment

In vitro: pure palbociclib (Carbosynth, cat. FA65120) was solubilized in HCl in equal molar ratio. The same HCl solution was used as vehicle control. Palbociclib HCl (Selleckchem, cat. S1116) was solubilized in water or DMSO as indicated. Vehicle controls for S1116 were HCl or acidified DMSO in equivalent molarity of palbociclib used in experimental treatments. In vivo: palbociclib was diluted/suspended in sodium lactate (50 mM, pH 4.0; adjusted with HCl) and administered by gavage at 140 mg/kg, 5 times/week. The control vehicle was sodium lactate (50 mM, pH 4.0). Lactic acid (Sigma, cat. 69785) was diluted in water to desired stock concentrations.

### Statistics

All statistical analyses were performed with PRISM 7 Software (GraphPad Software, La Jolla, CA, USA).

## Supplementary information

Supplemental Figures and Legends

## Data Availability

Source data (uncropped images of western blots) and accession numbers for the dataset analyzed herein are provided with this paper.
