# Supplementary material for: CDK4/6 inhibitors and the pRB-E2F1 axis suppress PVR and PD-L1 expression in triple-negative breast cancer
Source: Oncogenesis. 2023 May 26;12(1):29. doi: 10.1038/s41389-023-00475-1 (PMC10213015; doi:10.1038/s41389-023-00475-1)
Supplement: Supplementary file 1 — Supplemental Figures and Legends [file 41389_2023_475_MOESM1_ESM.pdf]

Supplemental Figures

CDK4/6-inhibitors and the pRB-E2F1 axis suppress PVR and PD-L1 expression in triple-negative breast cancer

Mariusz Shrestha[1, 2,*], Dong-Yu Wang[2], Yaacov Ben-David[3], Eldad Zacksenhaus[1,2,*]

[1] Department of Laboratory Medicine & Pathobiology, University of Toronto, Toronto, Ontario, Canada
[2] Toronto General Research Institute - University Health Network, 101 College Street, Max Bell Research Centre, Rm. 5R406, Toronto, Ontario, Canada, M5G 1L7
[3] The Key laboratory of Chemistry for Natural Products of Guizhou Province and Chinese Academic of Sciences, Guiyang, Guizhou, 550014, and State Key Laboratory for Functions and Applications of Medicinal Plants, Guizhou Medical University, Guiyang, 550025, China

Immune related hallmarks

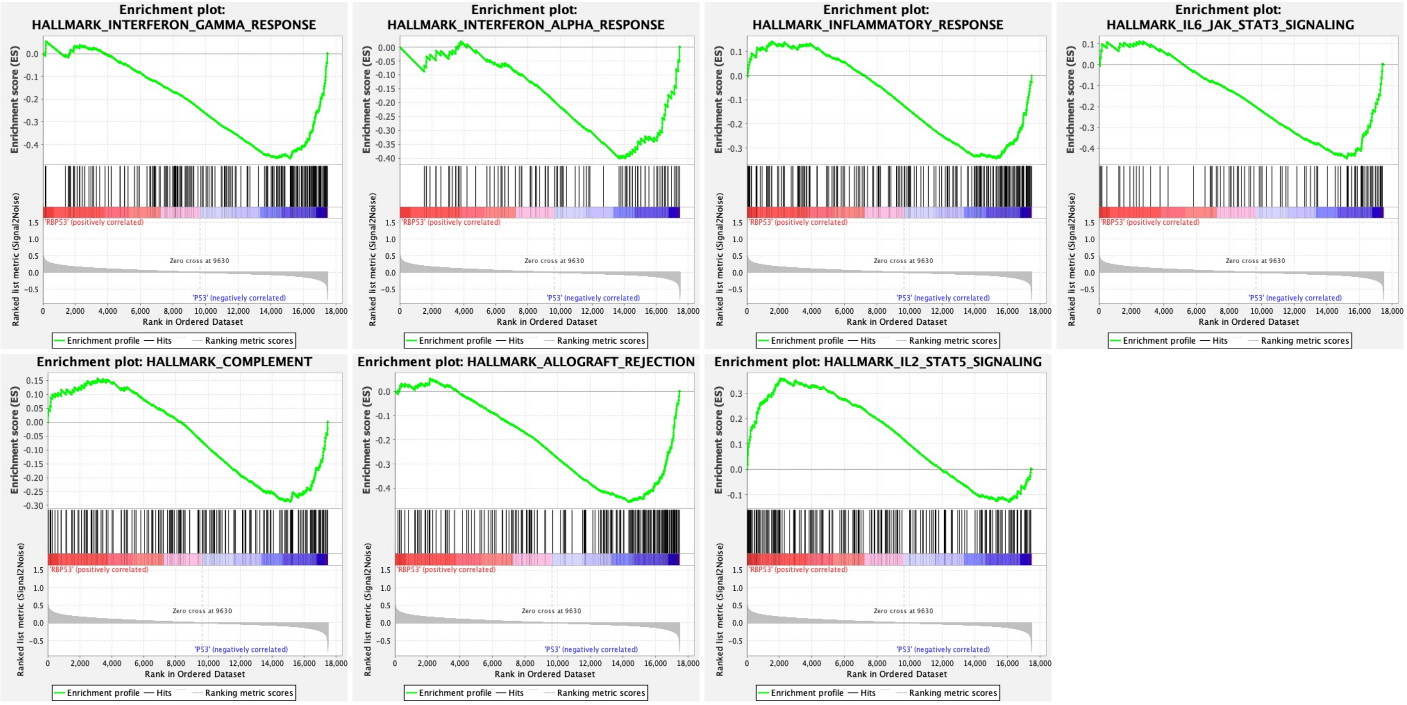

Other hallmarks

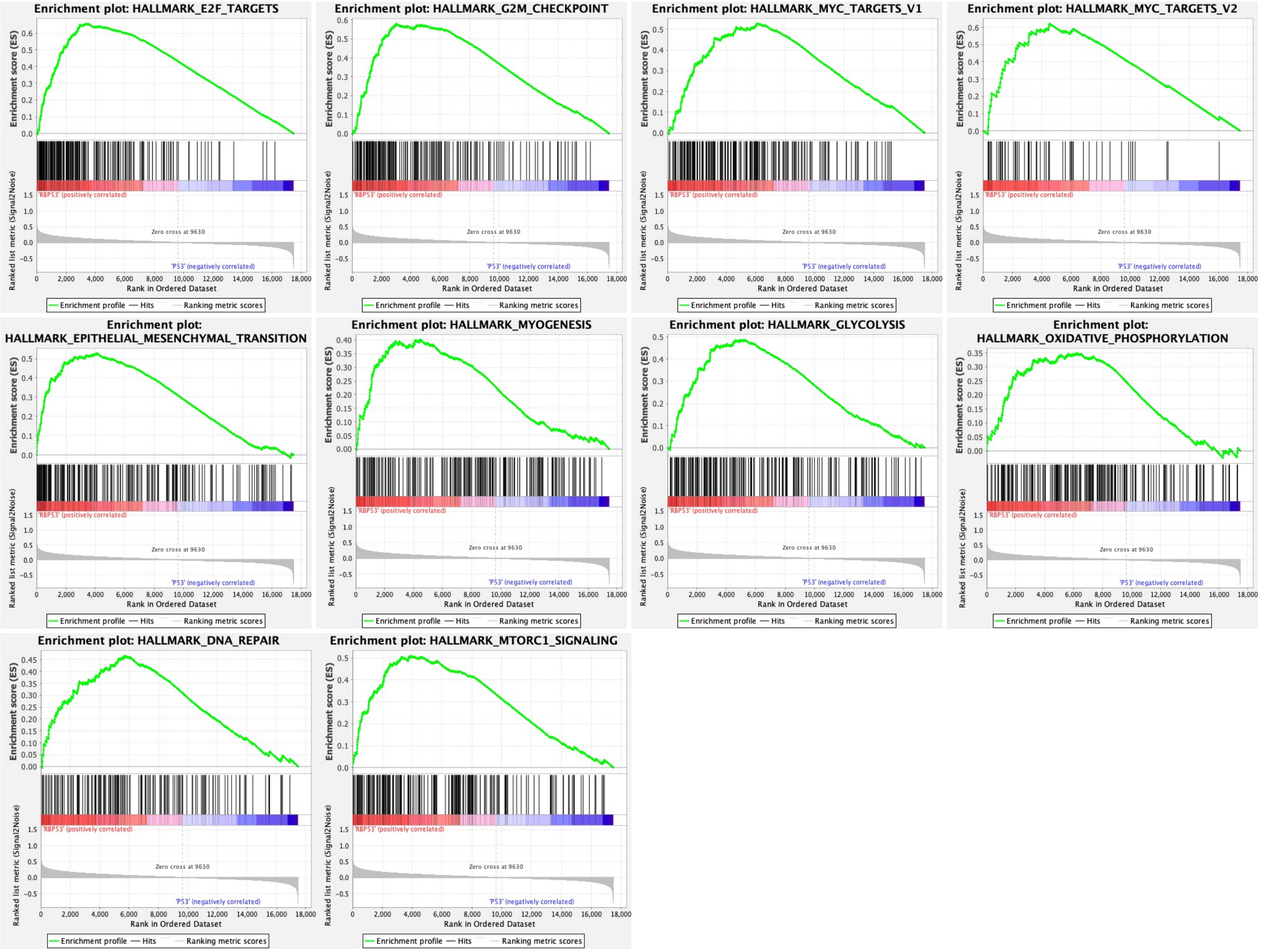

Figure S1 related to Figure 1A. GSEA enrichment plots for immune related and other hallmarks in Rb$^{\Delta}$p53$^{\Delta}$ versus p53$^{\Delta}$ mouse TNBC-like tumors.

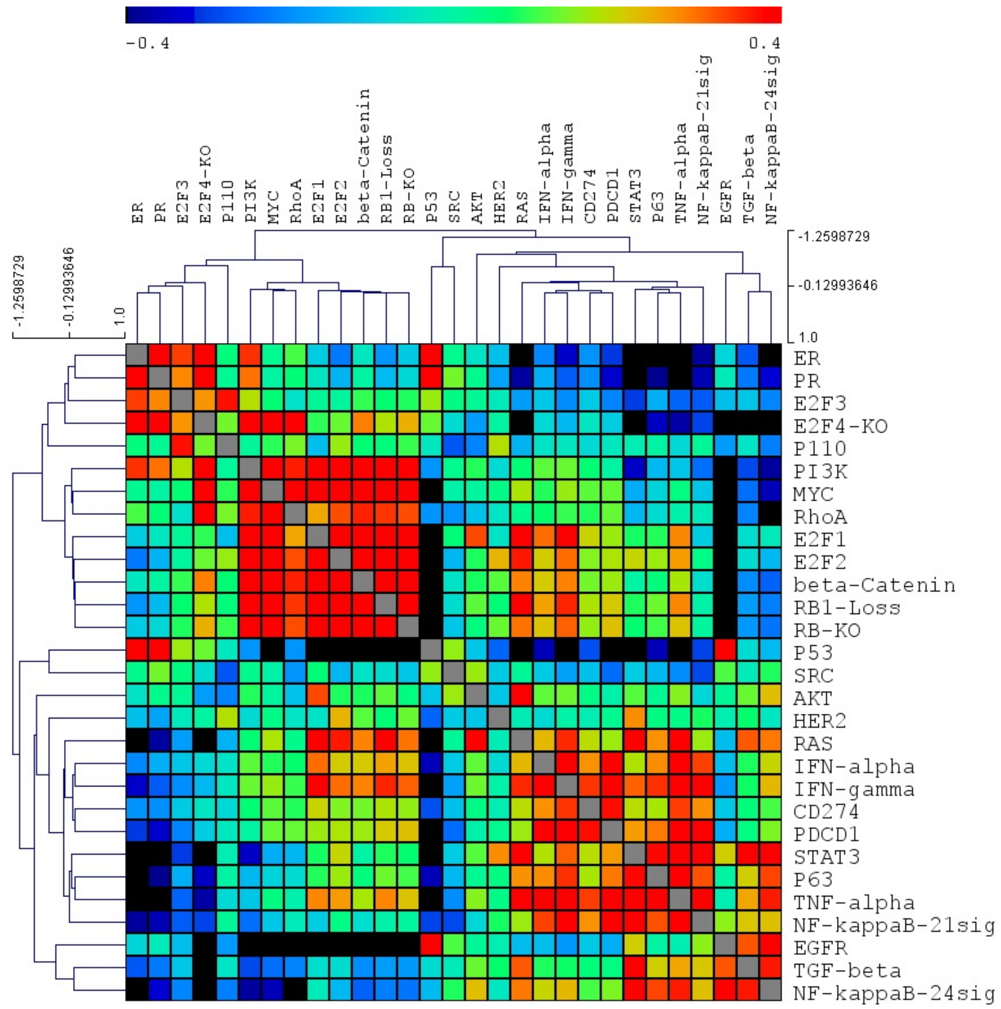
-0.4
0.4
ER
PR
E2F3
E2F4-KO
P110
PI3K
MYC
RhoA
E2F1
E2F2
beta-Catenin
RB1-Loss
RB-KO
P53
SRC
AKT
HER2
RAS
IFN-alpha
IFN-gamma
CD274
PDCD1
STAT3
P63
TNF-alpha
NF-kappaB-21sig
EGFR
TGF-beta
NF-kappaB-24sig
-1.2598729
-0.12993646
1.0

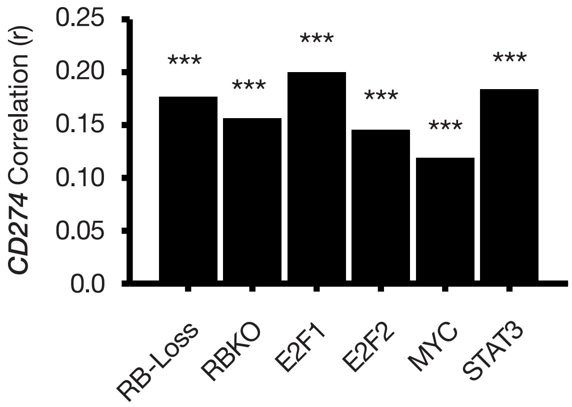
CD274 Correlation (r)
0.25
0.20
0.15
0.10
0.05
0.0
***
RB-Loss
RBKO
E2F1
E2F2
MYC
STAT3

Figure S2A

1302 BC

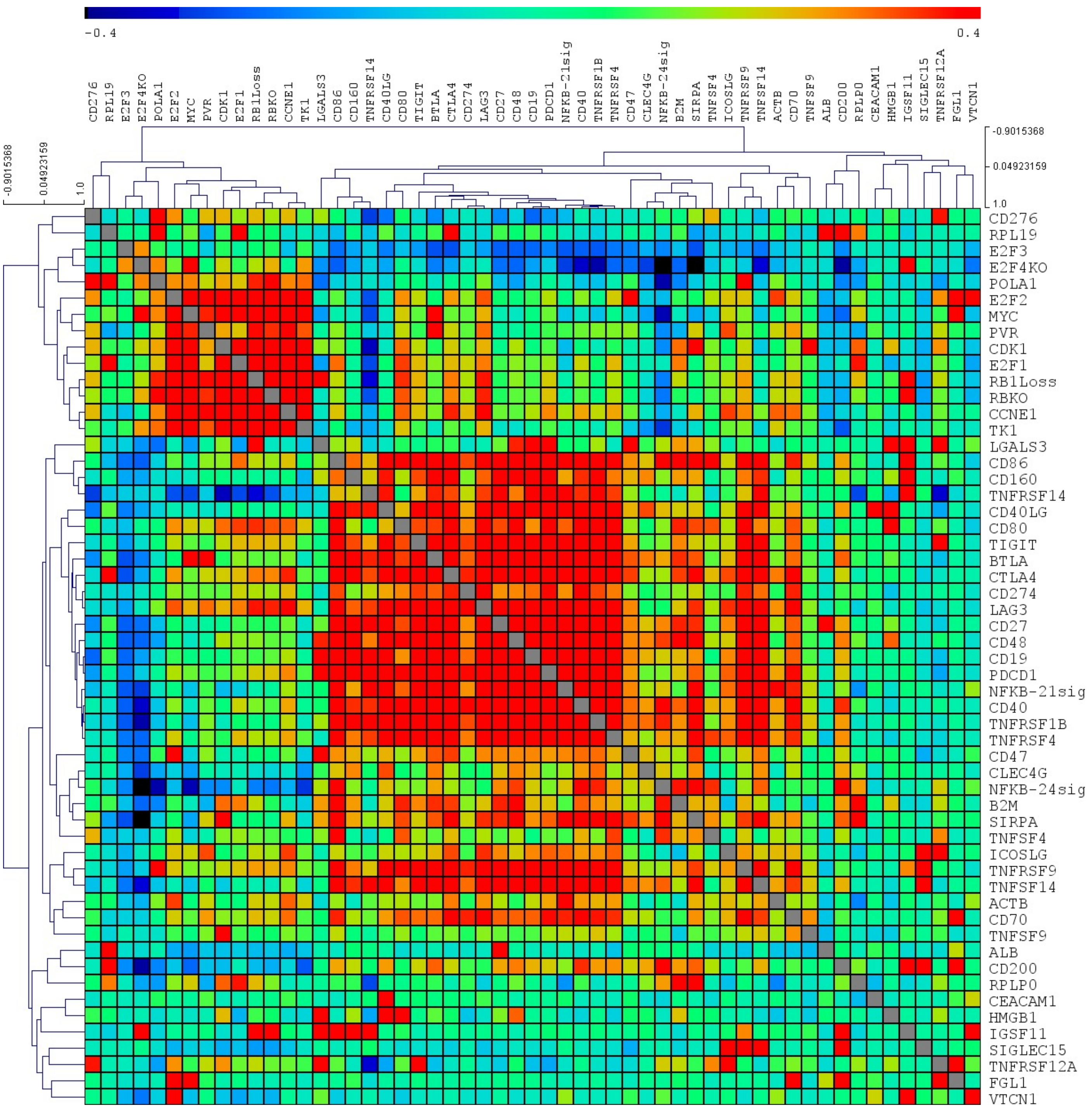

1302BC-r	r CCNE1	p-value CCNE1	r CDK1	p-value CDK1	r POLA1	p-value POLA1	r TK1	p-value TK1	r CD274	p-value CD274	r PVR	p-value PVR
E2F1	0.43754902	0.00000000	0.62218799	0.00000000	0.21592237	0.00000000	0.46472598	0.00000000	0.20017007	0.00000000	0.21849912	0.00000000
E2F2	0.61632620	0.00000000	0.65723760	0.00000000	0.26503897	0.00000000	0.61496852	0.00000000	0.14571406	0.00000013	0.38619790	0.00000000
E2F3	-0.12339639	0.00000799	0.08061747	0.00360408	0.09267289	0.00081417	0.08783369	0.00151178	-0.10471044	0.00015366	-0.10336187	0.00018693
E2F4KO	0.01866714	0.50095851	0.19773070	0.00000000	0.28420038	0.00000000	0.26321757	0.00000000	-0.04262970	0.12418579	0.05017454	0.07031737
RBKO	0.60411976	0.00000000	0.78035891	0.00000000	0.39482709	0.00000000	0.67338298	0.00000000	0.15688636	0.00000001	0.34883351	0.00000000
RB-loss	0.66365357	0.00000000	0.87815581	0.00000000	0.35870137	0.00000000	0.65425846	0.00000000	0.17711061	0.00000000	0.38321251	0.00000000
MYC	0.44745618	0.00000000	0.52173020	0.00000000	0.27612452	0.00000000	0.47403876	0.00000000	0.11906704	0.00001651	0.36235257	0.00000000
ACTB	0.31130604	0.00000000	0.06281878	0.02340448	0.14278028	0.00000023	0.21431975	0.00000000	0.08118876	0.00337242	0.19665750	0.00000000
ALB	-0.14754133	0.00000009	-0.11757550	0.00002107	-0.01328519	0.63198600	-0.10774724	0.00009800	-0.05212463	0.06006758	-0.11308445	0.00004320

Figure S2B

205 TNBC

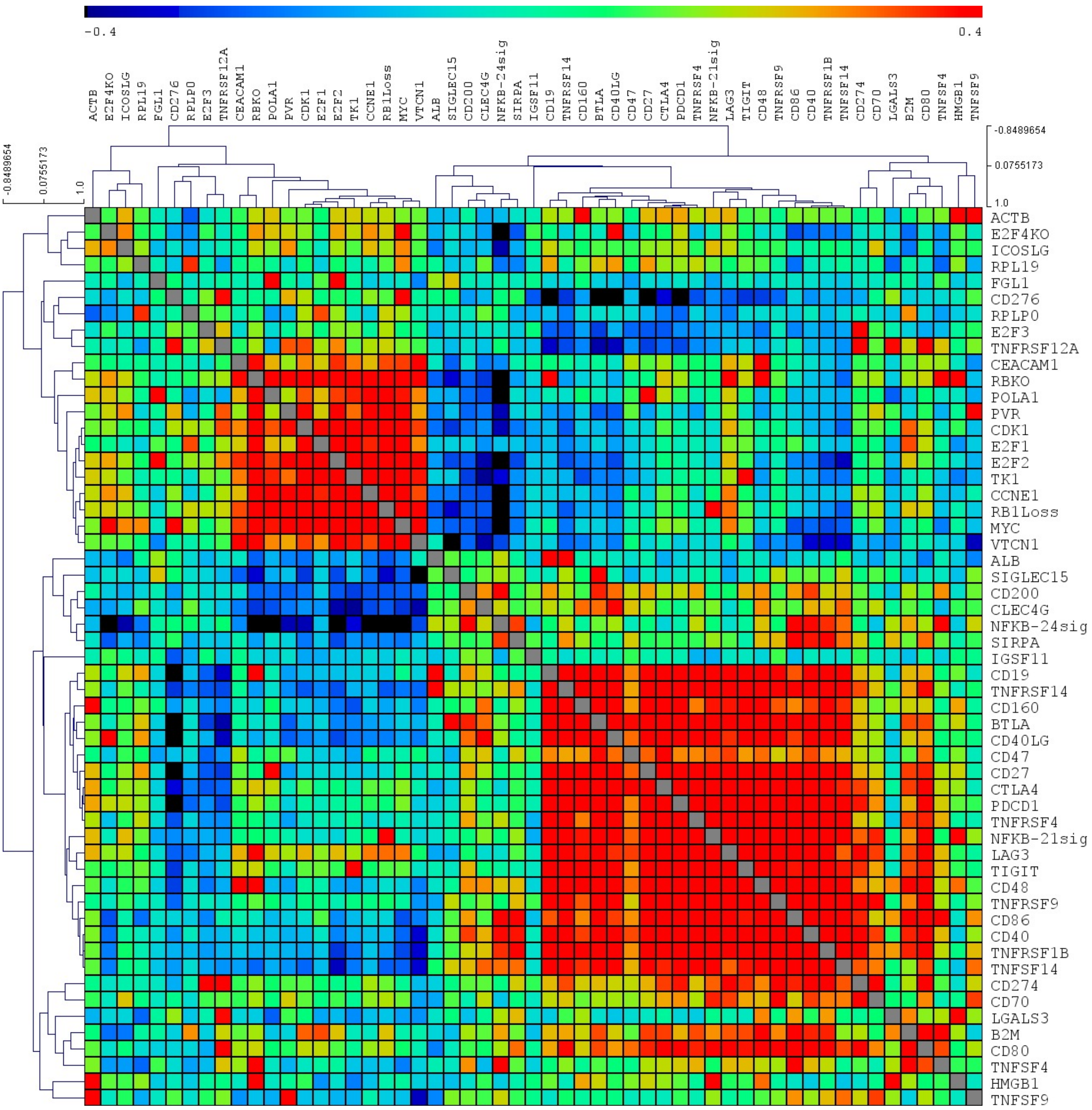

205TNBC-r	r CCNE1	p-value CCNE1	r CDK1	p-value CDK1	r POLA1	p-value POLA1	r TK1	p-value TK1	r CD274	p-value CD274	r PVR	p-value PVR
E2F1	0.36038036	0.00000011	0.59831353	0.00000000	0.22718697	0.00105335	0.50706623	0.00000000	0.05651071	0.42093153	0.21228786	0.00224446
E2F2	0.59069105	0.00000000	0.76244383	0.00000000	0.34194107	0.00000052	0.65967542	0.00000000	0.03429449	0.62543400	0.44636537	0.00000000
E2F3	0.01389375	0.84326301	0.14984362	0.03199785	-0.07254693	0.30126463	0.14188089	0.04242969	0.00248120	0.97183395	0.02208650	0.75326647
E2F4KO	0.26953306	0.00009302	0.15465382	0.02682162	0.22238386	0.00135152	0.18692031	0.00728297	-0.05383345	0.44330705	0.19911074	0.00420826
RBKO	0.64087515	0.00000000	0.77618316	0.00000000	0.45442144	0.00000000	0.62497011	0.00000000	0.15373467	0.02775120	0.45694087	0.00000000
RB-loss	0.66026530	0.00000000	0.86869715	0.00000000	0.40879521	0.00000000	0.64846962	0.00000000	0.12829678	0.06676071	0.45777391	0.00000000
MYC	0.56890488	0.00000000	0.60660914	0.00000000	0.36961299	0.00000005	0.48060181	0.00000000	0.01797319	0.79811763	0.47913144	0.00000000
ACTB	0.19452799	0.00519100	0.12508494	0.07393463	0.22449357	0.00121214	0.21190650	0.00228687	0.02733043	0.69728202	0.13372869	0.05592874
ALB	-0.21814455	0.00167692	-0.24393529	0.00042403	-0.14110366	0.04358590	-0.09083475	0.19522280	-0.09422128	0.17901769	-0.20555260	0.00310943

Figure S2C

Figure S2 related to Figure 1BC. (A) Top, a heat map showing correlation of gene expression with pathways and signatures in 1302 mix breast cancer (B-C) patient samples. Bottom, *CD274* correlation with known regulators (*MYC*, *STAT3*) and RB/E2Fs. (*P<0.05, **P<0.001, ***P<0.0001). (**B-C**) Expanded heat map correlation analysis of immune marker expression with gene expression, pathways and signatures in 1302 mix breast cancer (B-C) patient samples (B) or 205 TNBC patient samples (C).

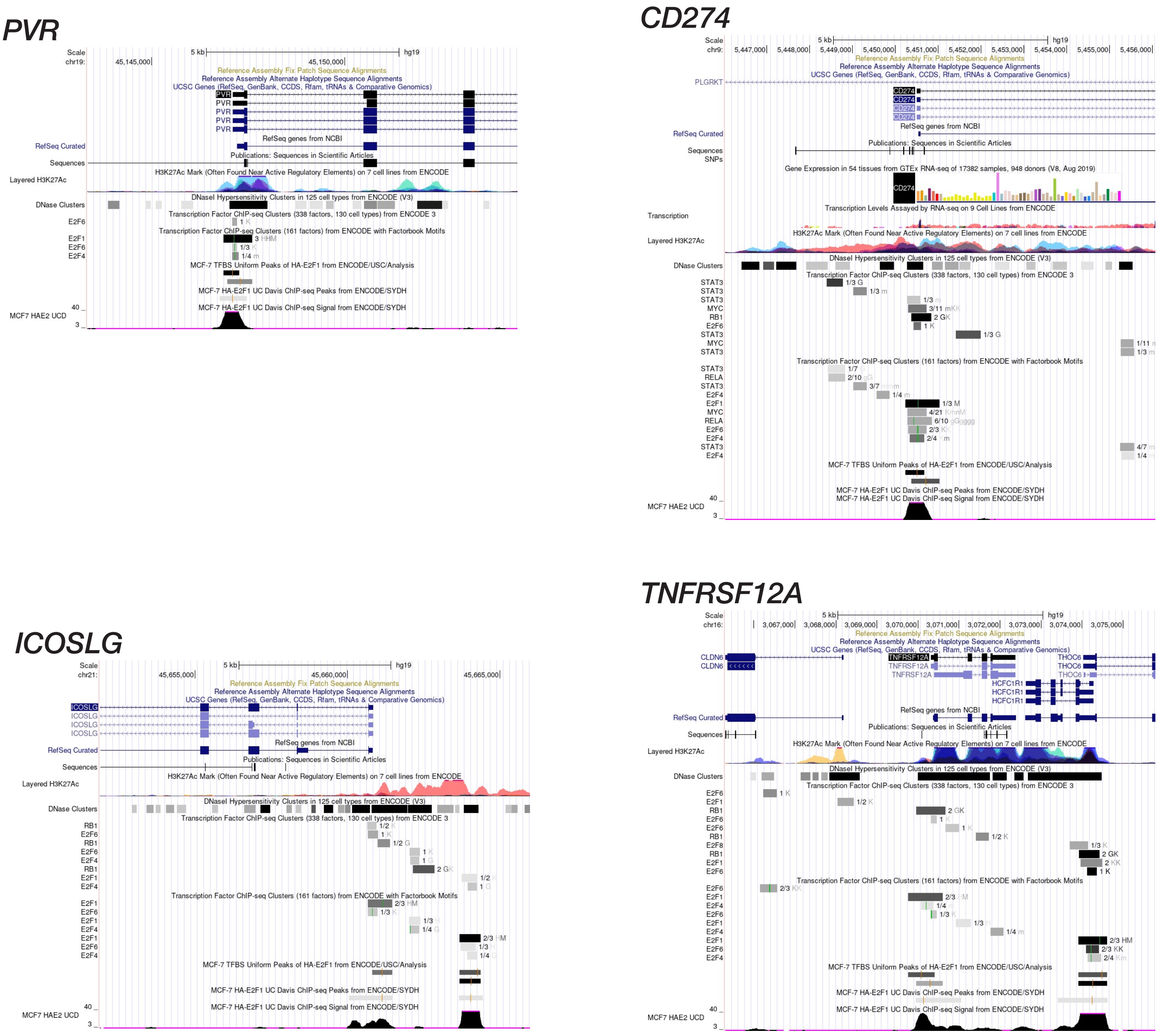
PVR
Scale
chr19:
5 kb
hg19
45,145,000
45,150,000
Reference Assembly Fix Patch Sequence Alignments
Reference Assembly Alternate Haplotype Sequence Alignments
UCSC Genes (RefSeq, GenBank, CCDS, Rfam, tRNAs & Comparative Genomics)
PVR
RefSeq genes from NCBI
RefSeq Curated
Publications: Sequences in Scientific Articles
Sequences
Layered H3K27Ac
H3K27Ac Mark (Often Found Near Active Regulatory Elements) on 7 cell lines from ENCODE
DNaseI Hypersensitivity Clusters in 125 cell types from ENCODE (V3)
DNase Clusters
Transcription Factor ChIP-seq Clusters (338 factors, 130 cell types) from ENCODE 3
E2F6
Transcription Factor ChIP-seq Clusters (161 factors) from ENCODE with Factorbook Motifs
E2F1
E2F6
E2F4
MCF-7 TFBS Uniform Peaks of HA-E2F1 from ENCODE/USC/Analysis
MCF-7 HA-E2F1 UC Davis ChIP-seq Peaks from ENCODE/SYDH
MCF-7 HA-E2F1 UC Davis ChIP-seq Signal from ENCODE/SYDH
MCF7 HAE2 UCD
40
3
CD274
Scale
chr9:
5 kb
hg19
5,447,000
5,448,000
5,449,000
5,450,000
5,451,000
5,452,000
5,453,000
5,454,000
5,455,000
5,456,000
PLGRKT
CD274
Gene Expression in 54 tissues from GTEx RNA-seq of 17382 samples, 948 donors (V8, Aug 2019)
SNPs
Transcription
Transcription Levels Assayed by RNA-seq on 9 Cell Lines from ENCODE
STAT3
MYC
RB1
E2F6
RELA
E2F4
E2F1
ICOSLG
Scale
chr21:
5 kb
hg19
45,655,000
45,660,000
45,665,000
ICOSLG
RB1
E2F6
E2F4
E2F1
TNFRSF12A
Scale
chr16:
5 kb
hg19
3,067,000
3,068,000
3,069,000
3,070,000
3,071,000
3,072,000
3,073,000
3,074,000
3,075,000
CLDN6
TNFRSF12A
THOC6
HCFC1R1
E2F8

Figure S3A

CD80

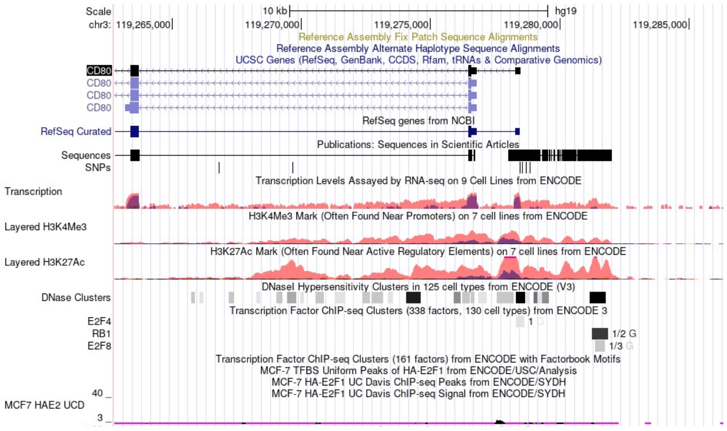

LAG3

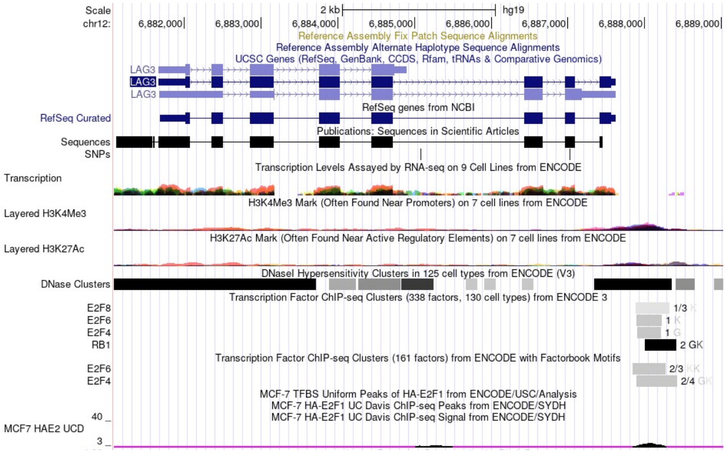

CTLA4

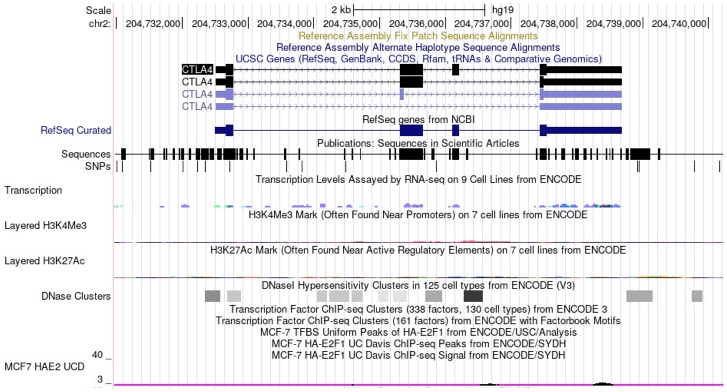

TIGIT

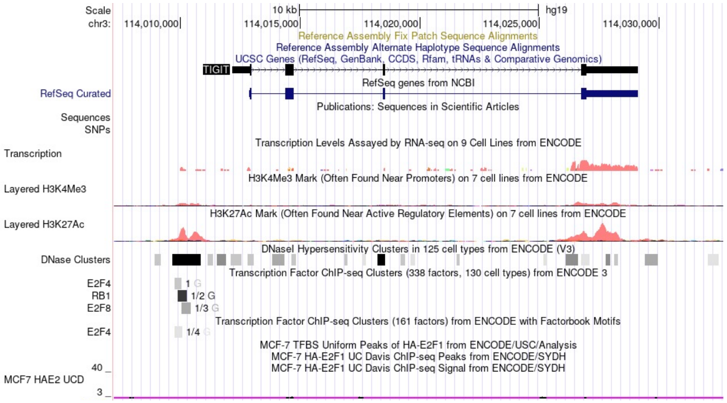

ALB

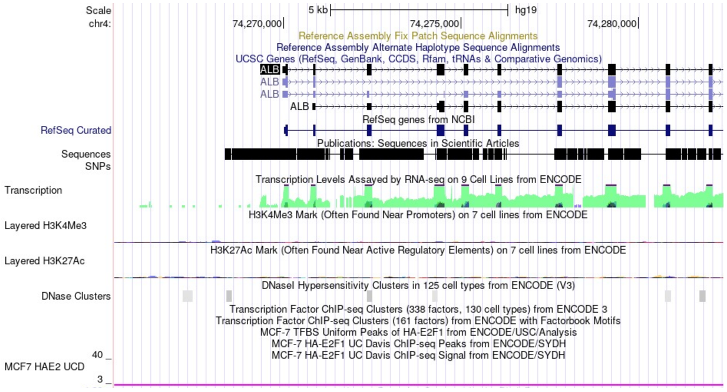

CCNE1

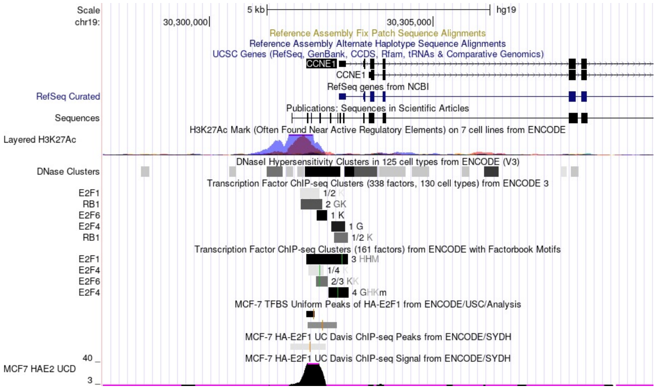

CDK1

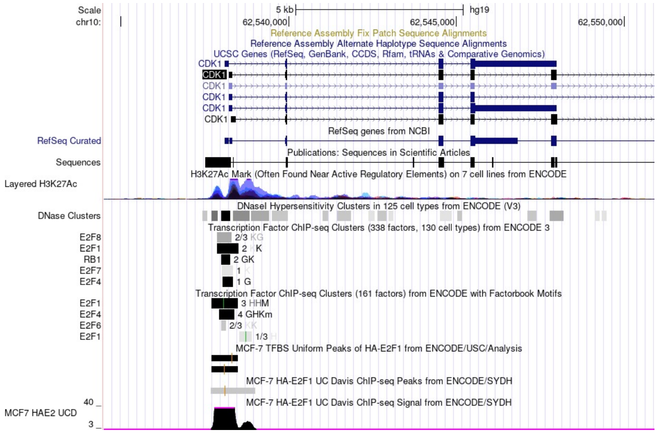

DHFR

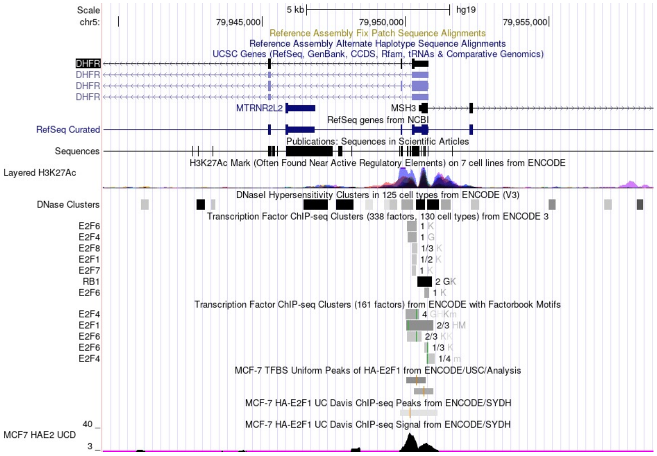

RBL1 (p107)

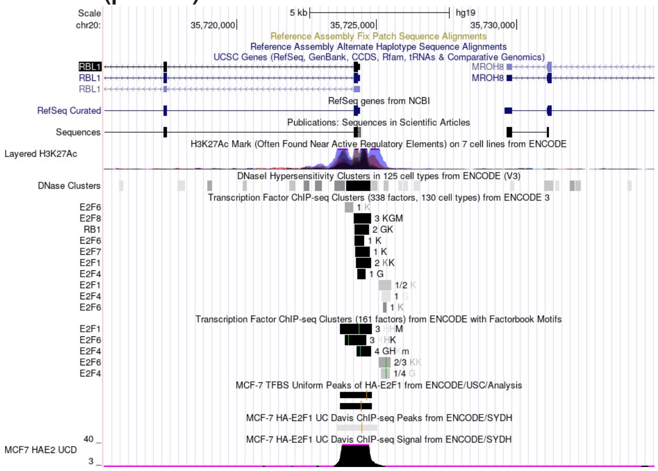

RB1

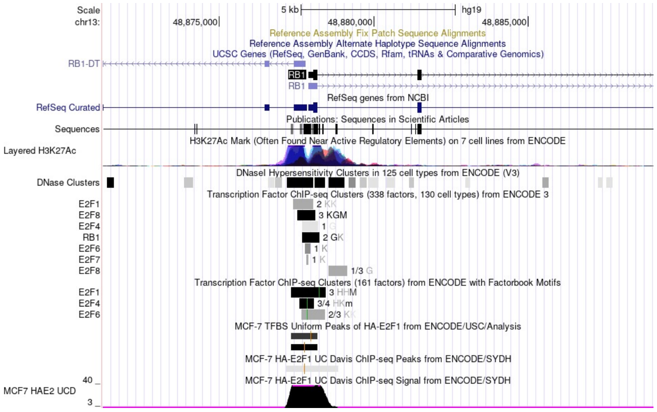

Figure S3C

STT3A

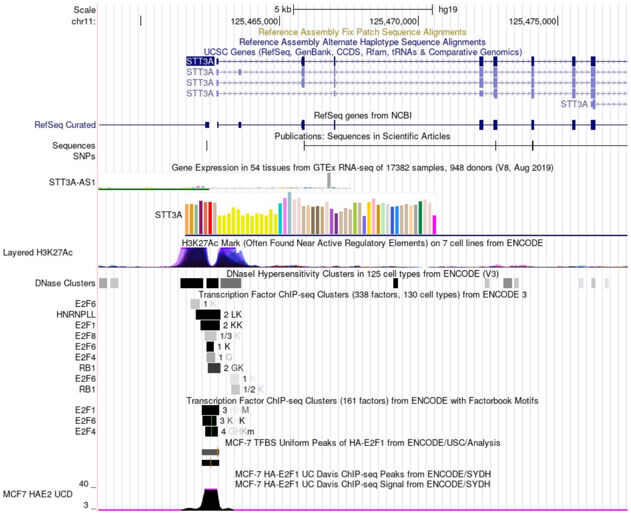

STT3B

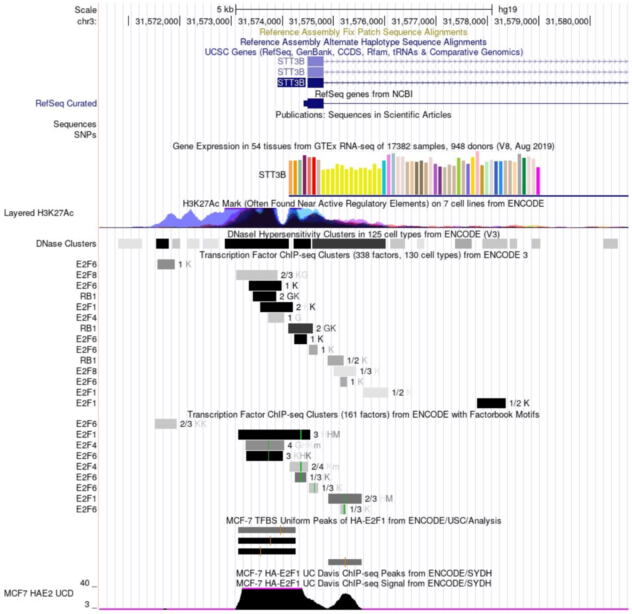

Figure S3 related to Figure 1D. ENCODE analysis of PVR, *CD247* (PD-L1 gene) and other immune-related genes for pRB, E2F and other transcription factors in various cell lines (bars) and in MCF7 breast cancer cells transduced with HA-E2F1 (bottom peaks). **(A)** RB and E2F recruitment to indicated immune marker promoters. **(B)** Immune marker genes that correlate with RB-loss and E2F1 signature expression but are negative for RB and E2F recruitment. Albumin promoter was also used as a negative control. **(C)** RB and E2F recruitment to *bona fide* RB-E2F regulated cell cycle genes, served as positive controls. **(D)** RB and E2F1 recruitment to promoters of genes that regulate PD-L1 glycosylation.

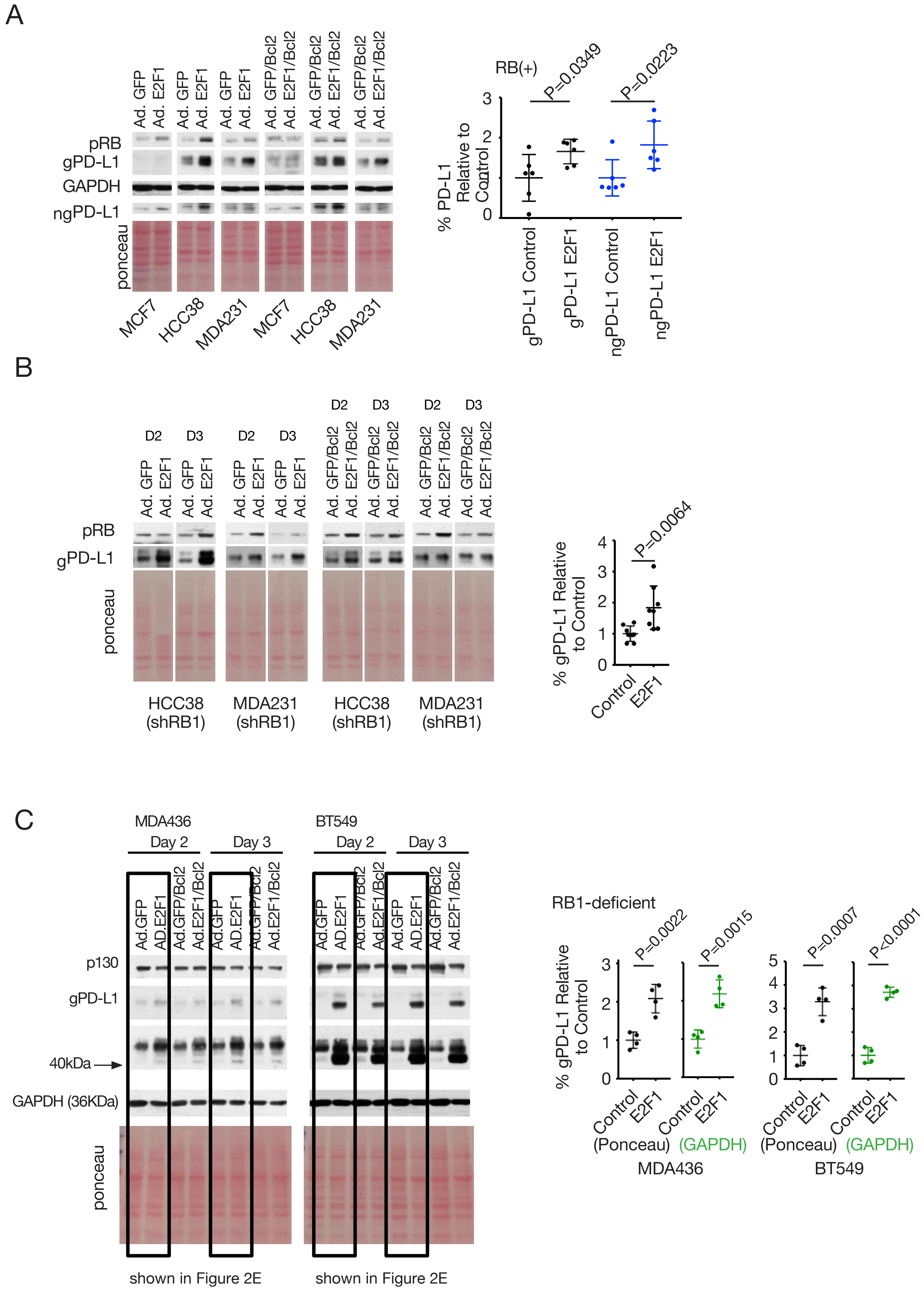
A
Ad. GFP
Ad. E2F1
Ad. GFP/Bcl2
Ad. E2F1/Bcl2
pRB
gPD-L1
GAPDH
ngPD-L1
ponceau
MCF7
HCC38
MDA231
RB(+)
P=0.0349
P=0.0223
% PD-L1 Relative to Control
gPD-L1 Control
gPD-L1 E2F1
ngPD-L1 Control
ngPD-L1 E2F1
B
D2
D3
HCC38 (shRB1)
MDA231 (shRB1)
P=0.0064
% gPD-L1 Relative to Control
Control
E2F1
C
MDA436
BT549
Day 2
Day 3
Ad.GFP
AD.E2F1
Ad.GFP/Bcl2
Ad.E2F1/Bcl2
p130
gPD-L1
40kDa
GAPDH (36KDa)
ponceau
shown in Figure 2E
RB1-deficient
P=0.0022
P=0.0015
P=0.0007
P<0.0001
% gPD-L1 Relative to Control
(Ponceau)
(GAPDH)
MDA436
BT549

Figure S4

Figure S4 related to Figure 2E. (A) Immunoblot analysis of PD-L1 in RB(+) breast cancer lines transduced with Ad.GFP, Ad.E2F1, Ad.GFP/Bcl2 or Ad.E2F1/Bcl2 (left). Cumulative quantification of PD-L1 expression (right). **(B)** Left, immunoblot analysis of PD-L1 in TNBC lines with stable depletion of *RB1* transduced with Ad.GFP, Ad.E2F1, Ad.GFP/Bcl2 or Ad.E2F1/Bcl2 for 2 (D2) or 3 (D3) days. Right, cumulative PD-L1 quantification. **(C)** Left, immunoblot analysis of PD-L1 in RB-deficient TNBC lines transduced with Ad.GFP, Ad.E2F1, Ad.GFP/Bcl2 or Ad.E2F1/Bcl2 for 2 (D2) or 3 (D3) days. Right, cumulative PD-L1 quantification in each cell line normalized by ponceau (black) or GAPDH (green). Both showing that over-expression of E2F1 induces PD-L1 in RB-depleted and RB-deficient TNBC cell lines.

A

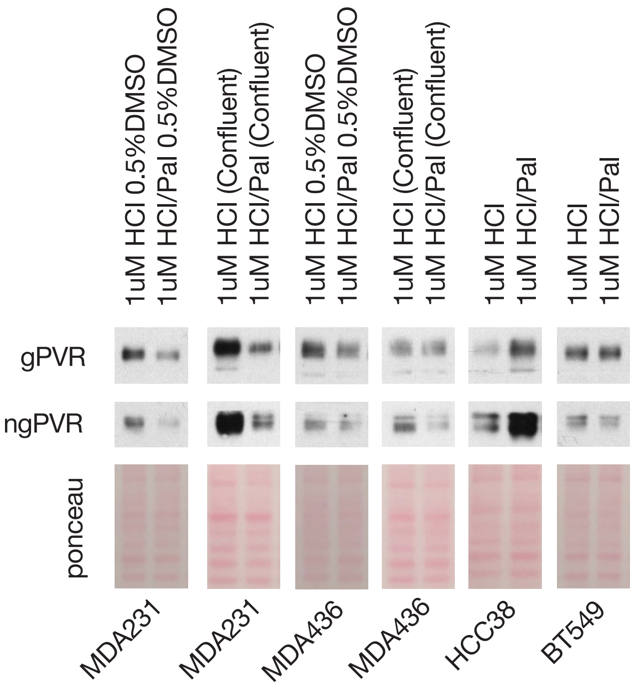

B

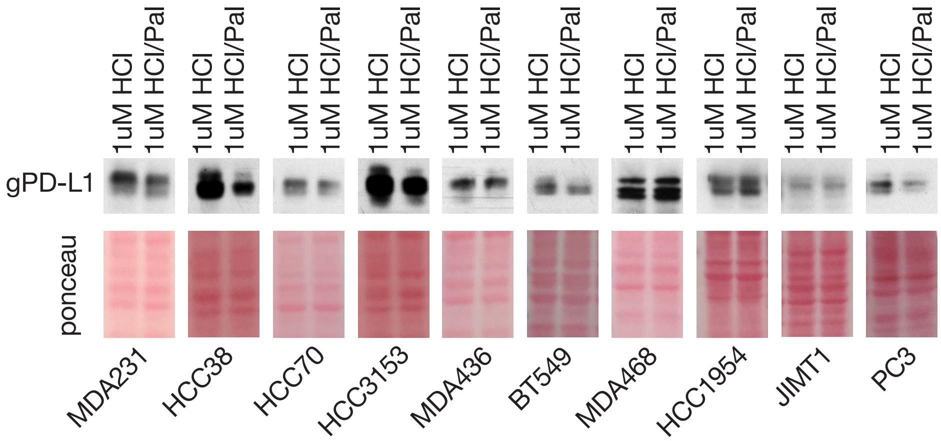

Figure S5 related to Figure 3. Immunoblot analysis showing the effect palbociclib treatment for 2 days on **(A)** PVR and **(B)** PD-L1 expression.

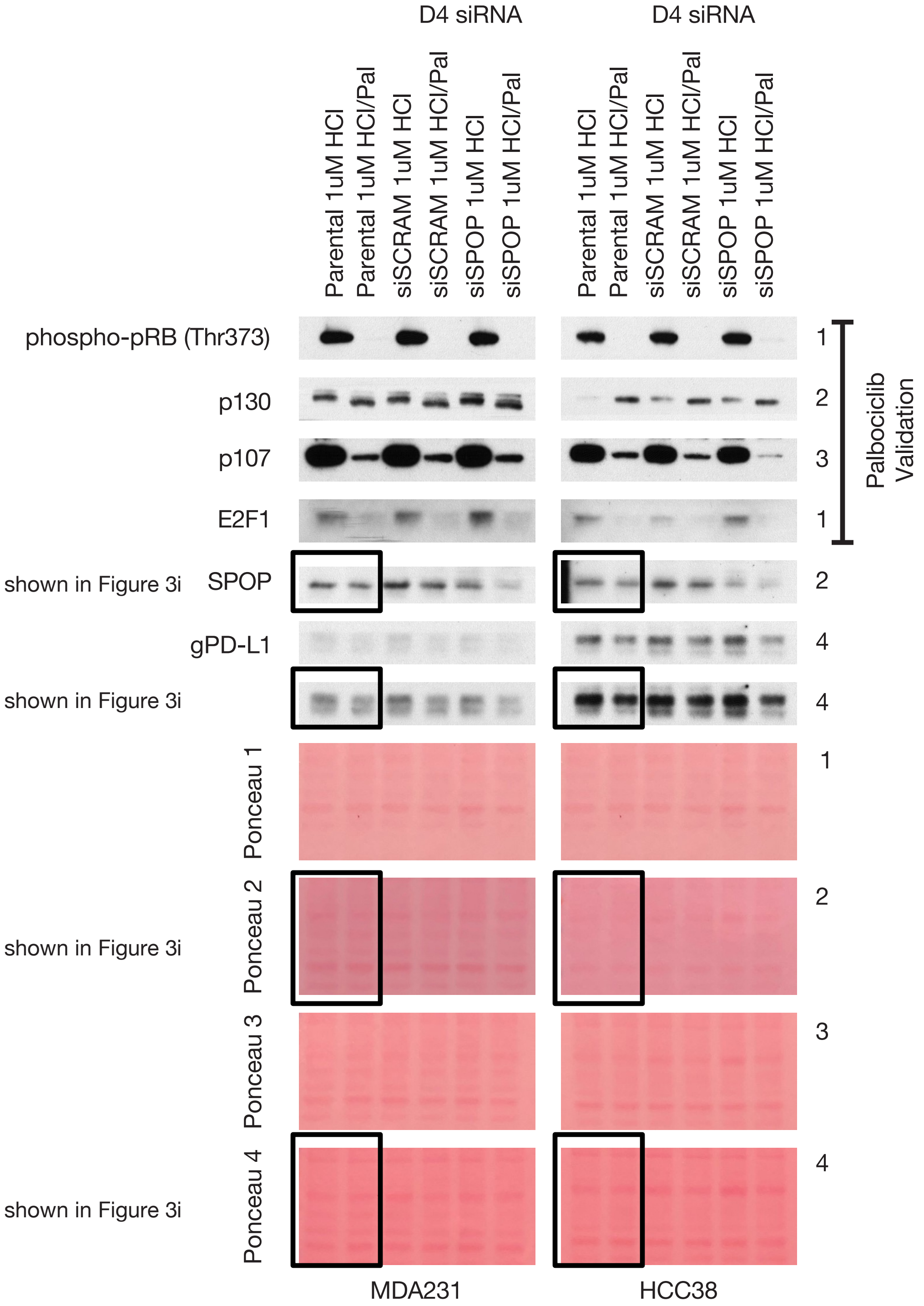
D4 siRNA
D4 siRNA
Parental 1uM HCl
Parental 1uM HCl/Pal
siSCRAM 1uM HCl
siSCRAM 1uM HCl/Pal
siSPOP 1uM HCl
siSPOP 1uM HCl/Pal
phospho-pRB (Thr373)
p130
p107
E2F1
shown in Figure 3i
SPOP
gPD-L1
shown in Figure 3i
Ponceau 1
shown in Figure 3i
Ponceau 2
Ponceau 3
shown in Figure 3i
Ponceau 4
1
2
3
1
2
4
4
1
2
3
4
Palbociclib Validation
MDA231
HCC38

Figure S6A

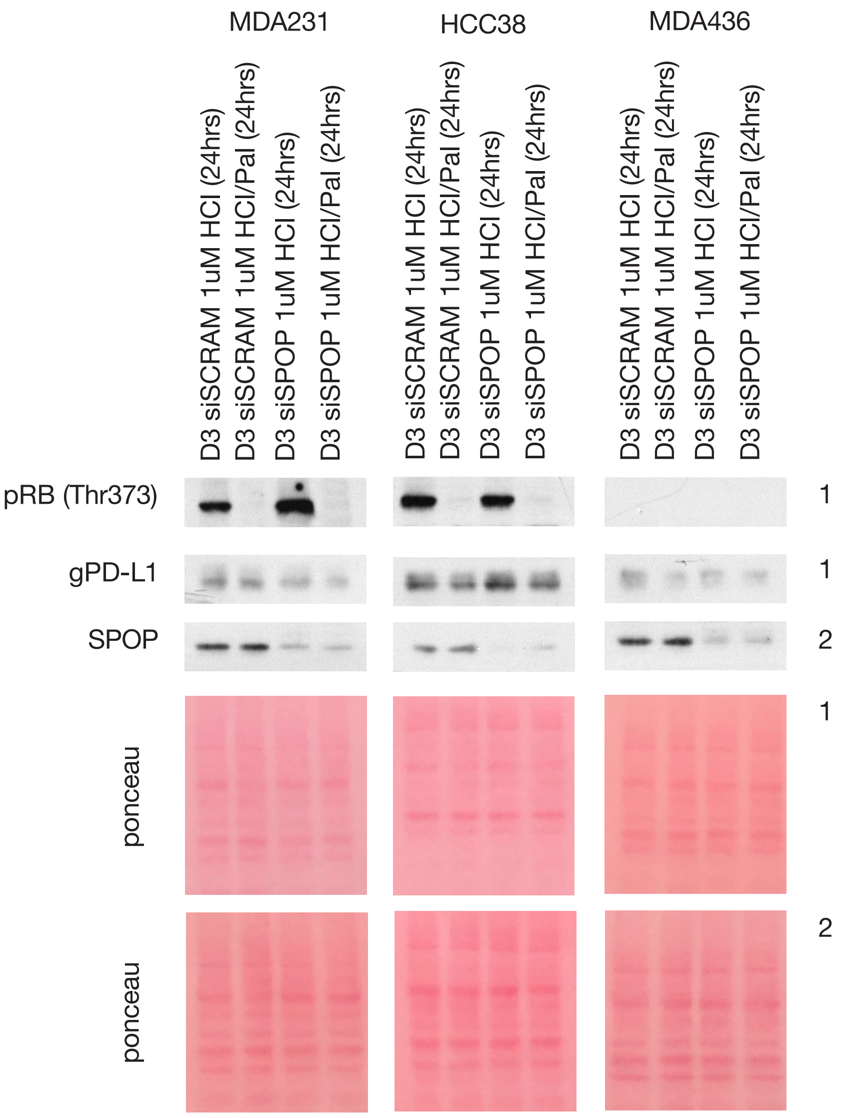

Figure S6 related to Figure 3H. (A) Immunoblot analysis of TNBC lines with/without transient knock-down of SPOP by RNAi after 4 days (D4) and treated with/without palbociclib for 2 days. **(B)** Immunoblot analysis of TNBC lines with/without transient knock-down of SPOP by RNAi after 3 days (D3) and treated with/without palbociclib for 24 hrs.

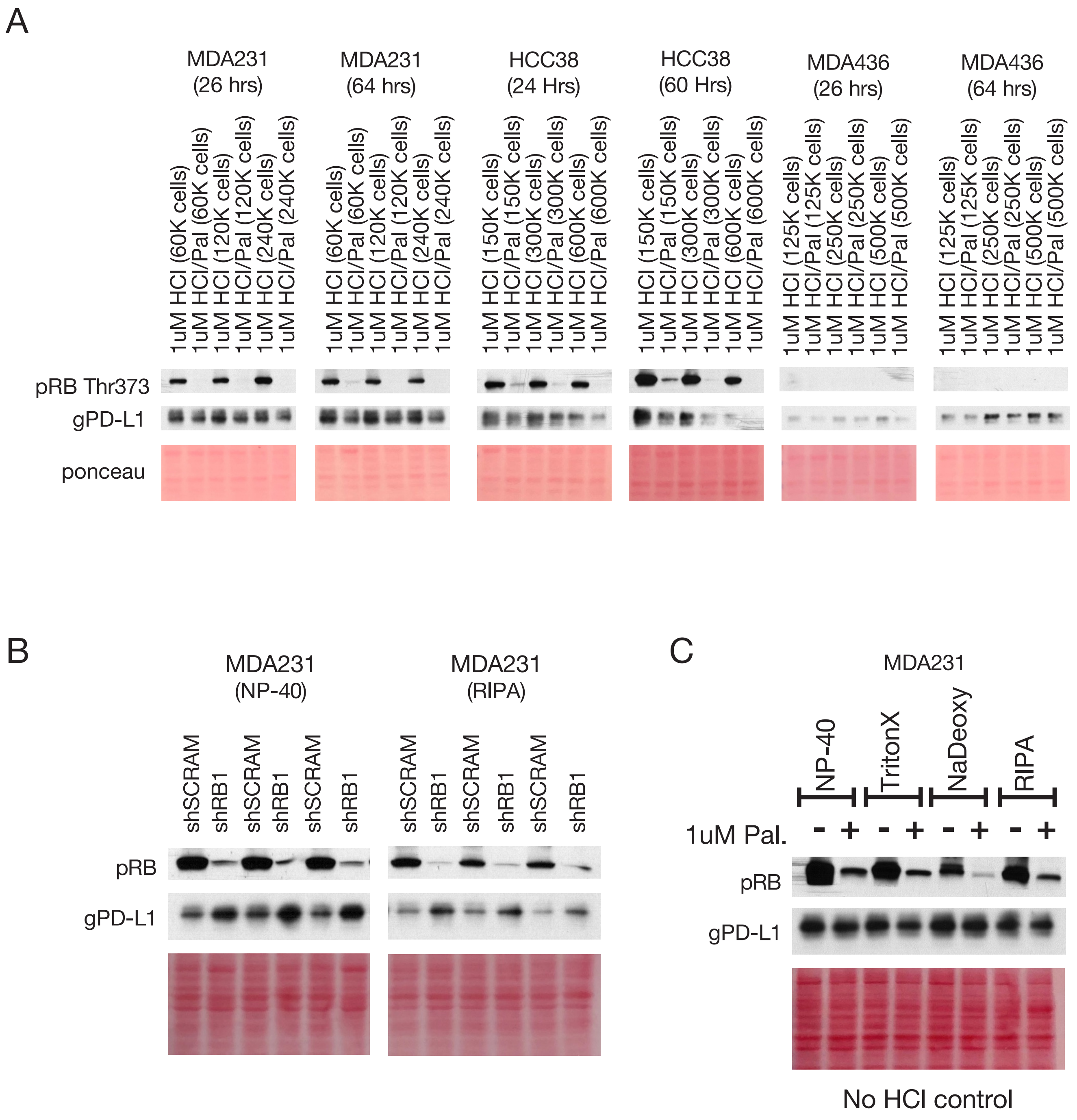

Figure S7 related to Figure 4A. (A) Immunoblot analysis of PD-L1 in TNBC lines seeded at various densities (in 6cm dishes) and treated with 1uM palbociclib for indicated durations. **(B)** Immunoblot analysis of PD-L1 in MDA-MB-231 cells with/without stable *RB1* knock-down lysed with NP-40 or RIPA buffer. **(C)** Immunoblot analysis of PD-L1 in MDA-MB-231 cells treated with palbociclib for 2 days and lysed with indicated buffers. Controls were used without adjusting for HCl concentration.

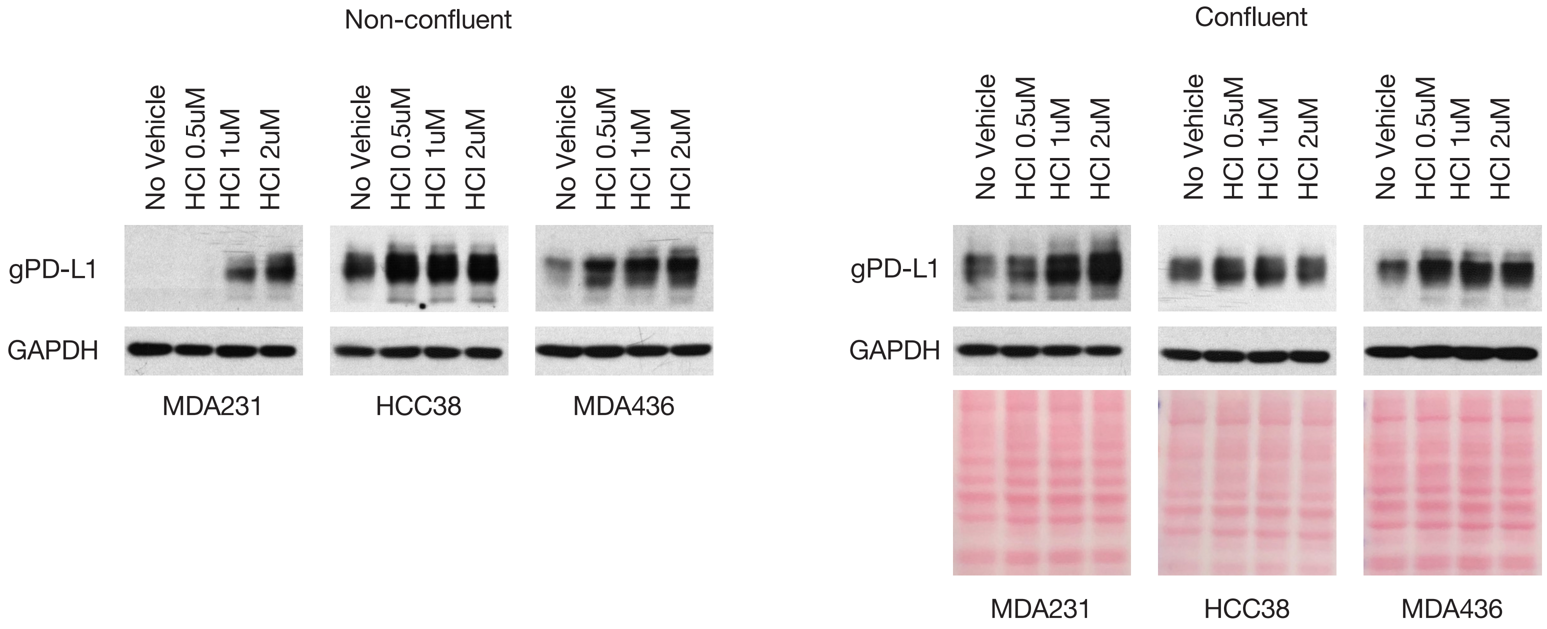

Figure S8 related to Figure 4B. Immunoblot analysis of TNBC lines treated with increasing concentrations of HCl for 2 days and lysed under non-confluent or confluent conditions.

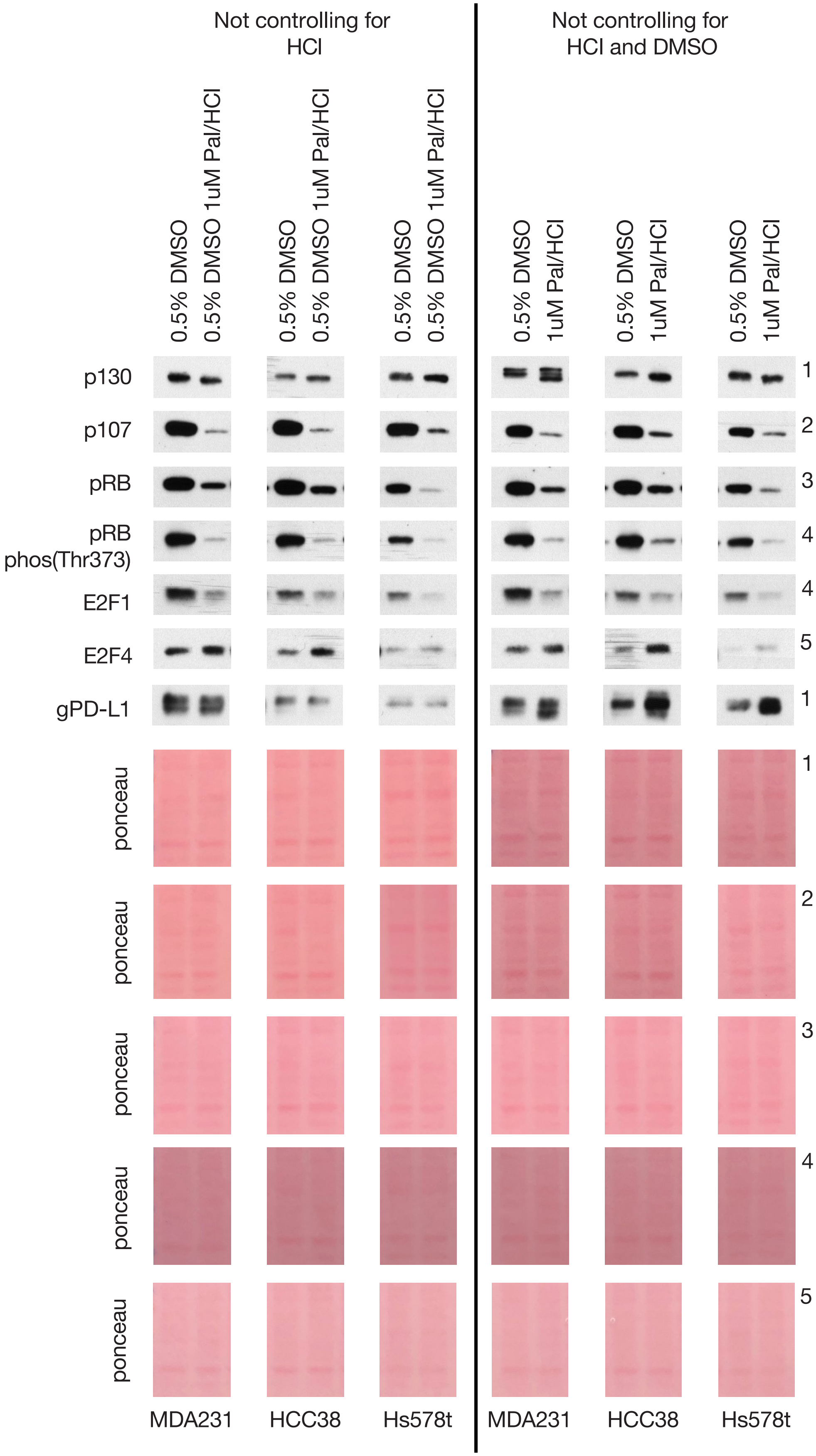

Figure S9 related to Figure 4B-C. HCl used to solubilize palbociclib and DMSO impacts gPD-L1 expression. Immunoblot analysis of PD-L1 in TNBC lines treated with palbociclib:HCl (Selleckchem-S1116, at equimolar ratio) for 2 days (D2) and controlled or not controlled for HCl or HCl/DMSO.
